# Spatiotemporal complexity patterns of resting‐state bioelectrical activity explain fluid intelligence: Sex matters

**DOI:** 10.1002/hbm.25162

**Published:** 2020-08-18

**Authors:** Joanna Dreszer, Marek Grochowski, Monika Lewandowska, Jan Nikadon, Joanna Gorgol, Bibianna Bałaj, Karolina Finc, Włodzisław Duch, Patrycja Kałamała, Adam Chuderski, Tomasz Piotrowski

**Affiliations:** ^1^ Centre for Modern Interdisciplinary Technologies Nicolaus Copernicus University Toruń Poland; ^2^ Faculty of Philosophy and Social Sciences Institute of Psychology, Nicolaus Copernicus University Toruń Poland; ^3^ Department of Informatics, Faculty of Physics, Astronomy, and Informatics Nicolaus Copernicus University Toruń Poland; ^4^ Faculty of Psychology University of Warsaw Warsaw Poland; ^5^ Department of Cognitive Science Institute of Philosophy, Jagiellonian University Krakow Poland

**Keywords:** fluid intelligence, frontoparietal network, multivariate multiscale sample entropy (mMSE), rsEEG, sex, spatiotemporal complexity patterns

## Abstract

Neural complexity is thought to be associated with efficient information processing but the exact nature of this relation remains unclear. Here, the relationship of fluid intelligence (*gf*) with the resting‐state EEG (rsEEG) complexity over different timescales and different electrodes was investigated. A 6‐min rsEEG blocks of eyes open were analyzed. The results of 119 subjects (57 men, mean age = 22.85 ± 2.84 years) were examined using multivariate multiscale sample entropy (mMSE) that quantifies changes in information richness of rsEEG in multiple data channels at fine and coarse timescales. *gf* factor was extracted from six intelligence tests. Partial least square regression analysis revealed that mainly predictors of the rsEEG complexity at coarse timescales in the frontoparietal network (FPN) and the temporo‐parietal complexities at fine timescales were relevant to higher *gf.* Sex differently affected the relationship between fluid intelligence and EEG complexity at rest. In men, *gf* was mainly positively related to the complexity at coarse timescales in the FPN. Furthermore, at fine and coarse timescales positive relations in the parietal region were revealed. In women, positive relations with *gf* were mostly observed for the overall and the coarse complexity in the FPN, whereas negative associations with *gf* were found for the complexity at fine timescales in the parietal and centro‐temporal region. These outcomes indicate that two separate time pathways (corresponding to fine and coarse timescales) used to characterize rsEEG complexity (expressed by mMSE features) are beneficial for effective information processing.

## Graphical abstract

Neural complexity is thought to be associated with efficient information processing but the exact nature of this relation remains unclear. Here, the relationship of fluid intelligence (*gf*) with the resting‐state EEG (rsEEG) complexity over different timescales and different electrodes was investigated. Partial least square regression analysis revealed that mainly predictors of the rsEEG complexity at coarse timescales in the fronto‐parietal network and the temporo‐parietal complexities at fine timescales were relevant to higher *gf*.

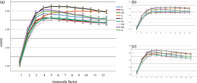

## 1 INTRODUCTION

The human brain is perceived as a complex network composed of interconnected regions that constantly process and integrate information with coherent temporal dynamics (Honey et al., 2009; Sporns, Chialvo, Kaiser, & Hilgetag, 2004; van den Heuvel, Stam, Kahn, & Hulshoff Pol, 2009). It is thought that the moment‐to‐moment variability of functional network states reflects important information about the topology and dynamics of brain networks across spatial and temporal scales (Faisal, Selen, & Wolpert, 2008; Garrett et al., 2013; Miskovic, Owens, Kuntzelman, & Gibb, 2016; Pincus, 1991; Tognoli & Kelso, 2014). This particularly refers to the situation when there is no explicit task and a subject is instructed to relax and not to “think about anything special” (Cabral, Kringelbach, & Deco, 2014; Deco & Corbetta, 2011; Deco, Jirsa, et al., 2013). From this point of view, the brain at rest may be modeled as a multistable dynamical system transitioning among diverse network states. An insight into brain spontaneous fluctuation patterns within particular networks may be obtained using external time‐series observations, for example, electroencephalogram (EEG) or magnetoencephalogram (MEG) signals (Kelso, 1995; Stam, 2005). A key property of a dynamical system that can be inferred from such observable time‐series data is its complexity understood as the richness of information conveyed by system state transitions in the spatiotemporal domain (Costa, Goldberger, & Peng, 2005; Frigg & Werndl, 2011; Pincus, 1991). The concept of complexity in this sense is intrinsically linked with both the information‐theoretic notion of entropy and the concept of entropy of a dynamical system (Frigg & Werndl, 2011).

Indeed, back in the '60s, Pinneo (1966) noted that it is not necessarily the stimulus‐induced “phasic” neural activity, but rather the baseline, task‐independent “tonic” activity that enabled effective neural functioning. Spontaneous low‐frequency fluctuations of blood‐oxygen‐level dependent signals investigated using functional magnetic resonance imaging (fMRI) have been found to be highly structured (Biswal et al., 2010; Damoiseaux et al., 2006). Specifically, they changed synchronically in functionally separate regions within the networks subserving critical sensory and cognitive functions (Allen et al., 2011; Damoiseaux et al., 2006). One such brain network is the frontoparietal network (FPN) that plays an important role in cognitive control (Gordon et al., 2018). Several studies indicate that individual differences in the characteristics of spontaneous spatiotemporal fluctuations, pronounced in the FPN (Finn et al., 2015; Gratton et al., 2018; Mueller et al., 2013), might be related to intellectual abilities (Finn et al., 2015).

Spatiotemporal patterns of the resting brain's activity appear to change much more rapidly than they could be detected using techniques with a poor temporal resolution, for example, fMRI (Baker et al., 2014; Deco & Corbetta, 2011; Siegel, Donner, & Engel, 2012). Moreover, the ambiguity of the physiological sources of the hemodynamic signal limits the insight into the mechanisms governing the relationship between activity at rest and activity associated with tasks, and consequently with behavioral results (Fox & Raichle, 2007). Therefore, more appropriate methods, for example, EEG or MEG should be applied to investigate these fast network state transitions; which have already been used to demonstrate that the dynamic organization of spontaneous interactions between networks contribute to more efficient communication in the brain (de Pasquale, Della Penna, Sporns, Romani, & Corbetta, 2016; Liu, Farahibozorg, Porcaro, Wenderoth, & Mantini, 2017; Siegel et al., 2012). Since it has been hypothesized that such fast information transfer efficiency is linked to intellectual abilities, the present study tested this assumption by exploring the resting EEG (rsEEG) signal complexity and its relation to fluid intelligence (*gf*).

*Gf* is defined as the ability to solve novel problems by adaptive reasoning without resorting to acquired knowledge or referring to previous experience (Cattell, 1963; Carroll, 1993, Horn & Cattell, 1967; for review: McGrew, 2009). Fluid intelligence understood in this way can then be defined as a facet of intellect related to the capacity to process and integrate information (Deary, Penke, & Johnson, 2010; Duncan et al., 2000; Jensen, 1998; Jung & Haier, 2007; Luders, Narr, Thompson, & Toga, 2009) by flexible transitions between network states (Barbey, 2018; Colom, Jung, & Haier, 2006; Colom, Karama, Jung, & Haier, 2010; Gordon et al., 2018). The greatest dynamic flexibility, which is thought to be crucial for adaptation to environmental demands (Friston, 2011), has been found in networks closely related to fluid intelligence (mainly the FPN) (Gu et al., 2015). The FPN has been labeled in other contexts as the executive control network (Dosenbach et al., 2006) or considered as a part of a larger control network comprising the cingulo‐opercular network and the dorsal attention network (Gordon et al., 2018). Thus, the role of the FPN in fluid reasoning ability might be identified with cognitive control shaping neural network dynamics, supporting specific cognitive functions essential to solve a novel challenging task (Barbey, 2018).

Existing evidence on the FPN as the neuroanatomical foundation of intelligence (the parieto‐frontal integration theory of intelligence, P‐FIT, Jung & Haier, 2007) is mainly derived from fMRI and PET studies focused on task‐related activations (Colom et al., 2010; Jung & Haier, 2007), whereas task‐free outcomes remain inconsistent (e.g., Dubois, Galdi, Paul, & Adolphs, 2018; Ferguson, Anderson, & Spreng, 2017; Finn et al., 2015; Hearne, Mattingley, & Cocchi, 2016; Santarnecchi, Emmendorfer, & Pascual‐Leone, 2017). This inconsistency might be partly caused by the use of different methods to evaluate intelligence and resting‐state brain activity. Currently, the whole‐brain network connectivity and interactions at rest are thought to be involved in *gf* (Ferguson et al., 2017; Finn et al., 2015; Hearne et al., 2016; M. Li et al., 2019). Specifically, the connectivity between the right hippocampus and the medial prefrontal cortex (R. Li, Zhang, Wu, Wen, & Han, 2020), the interactions of the lateral prefrontal cortex with other networks (Cole, Ito, & Braver, 2015) or the connections between the FPN, default mode, salience and motor‐sensory network (M. Li et al., 2019) have been linked to fluid intelligence. It is worth noting, however, that the FPN interactions are still found to be most predictive for *gf* level (Ferguson et al., 2017; Finn et al., 2015; M. Li et al., 2019).

Fink and Neubauer's team extensively explored the brain substrates of intelligence in the context of the neural efficiency hypothesis and found more focused activations during performance on various problem‐solving tasks in highly‐intelligent—compared to average‐intelligent persons (Neubauer & Fink, 2009a, 2009b). These studies also showed that sex mattered when the relationship between intellectual abilities and task‐related neural activity was explored. For example, Neubauer and Fink (2003) found that a higher level of intellectual abilities coexisted with more focused activation during intelligence test performance only in male subjects. According to the authors' best knowledge, in the context of *gf*, the role of sex in resting‐state neural activity (including the specificity of the FPN) has not been well recognized to date.

Previous studies have revealed an inconsistency in the relationship between individual alpha frequency at rest and intelligence suggesting either a positive association (Anokhin & Vogel, 1996; Grandy et al., 2013) or no relation (Jaušovec & Jaušovec, 2000; Posthuma, Neale, & Boomsma, 2001). These contradictory results might be due to different methods used to assess intelligence or uncontrolled the sex effect (Pahor & Jaušovec, 2017). Furthermore, persons with higher intelligence, relative to those with lower IQ, demonstrated reduced alpha power (Jaušovec, 1997), enhanced alpha power (Jaušovec, 1996), or no differences were observed (Jaušovec, 2000).

Recently, intelligence is being considered with a reference to a small‐world brain organization (Colom et al., 2006; Langer et al., 2012; Thatcher, Palmero‐Soler, North, & Biver, 2016; van den Heuvel et al., 2009), defined as high local clustering and the short path length of the network comprising the nodes interconnected by the lines or edges with adjacent nodes (Watts & Strogatz, 1998). It has been discovered that fluid intelligence positively correlated with enhanced small‐world organization of rsEEG higher alpha band (10.5–12 Hz) over the right posterior area whereas a negative relationship was observed between *gf* and local connectivity in the frontal cortex and posterior cingulate gyrus (Langer et al., 2012). Thatcher et al. (2016) found a negative relation of intelligence with the magnitude of information flow, obtained from rsEEG data. Furthermore, in this study, the greatest differences between the groups characterized by low and high IQ were determined for the electrodes separated by long distances. Above outcomes have been explained in terms of small‐world brain topology: a higher intelligence level coexists with more efficient local information processing that produces less demands from more‐distant nodes of the network. On the other hand, several resting‐state fMRI studies emphasized a significance of long‐distance connections in intellectual behavior (Colom et al., 2006; Santarnecchi, Galli, Polizzotto, Rossi, & Rossi, 2014; van den Heuvel et al., 2009). Specifically, there are evidence on a strong positive relationship between *gf* and global communication efficiency in the brain (van den Heuvel et al., 2009) and also moderately weak long‐distance connections are thought to explain the most of variance in intelligence tests scores (Santarnecchi et al., 2014). All these studies have raised the importance of taking into account both local and global information transfer while investigating the neural substrates of fluid intelligence.

The dynamic flexibility of a network may be evaluated in terms of neural complexity, understood as a product of different kinds of nontrivial interactions emerging from the coexistence of synchronized and desynchronized subsystems (Ibáñez‐Molina & Iglesias‐Parro, 2016), the balance between functional segregation and integration processes (Tononi, Sporns, & Edelman, 1994; Deco, Ponce‐Alvarez, et al., 2013), and the neural noise (Cabral et al., 2014; Cabral, Kringelbach, & Deco, 2017). Neural complexity is conceived as the randomness of temporal fluctuations' patterns of brain activity within a region or network (McDonough & Nashiro, 2014). These patterns are thought to reflect the dynamic flexibility of the brain network, that is, its capacity to change rapidly over time in order to work in the most efficient manner. In this study, the complexity of the rsEEG (mainly in FPN, but not limited to its areas) was assessed using an extension of sample entropy (SampEn) measure that enabled examination of the complexity of resting neural network state transitions in the spatiotemporal domain.

SampEn is related to the Kolmogorov–Sinai entropy (KS entropy) of a dynamical system and has been introduced specifically for the analysis of nonstationary physiological signals (Richman & Moorman, 2000). Furthermore, for a multivariate signal such as EEG, the multivariate MSE (mMSE) proposed by Looney, Adjei, and Mandic (2018) examined complexity across both time and space (channels). The vector‐valued complexity profiles of a signal obtainable using MSE or mMSE have been demonstrated to contain more information on the complexity of the underlying system than the SampEn measure alone. It should also be noted that complex dynamics typically involve structures across temporal scales, from fine‐scales to coarse‐scales (Ahmed & Mandic, 2011; Catarino, Churches, Baron‐Cohen, Andrade, & Ring, 2011; Costa, Goldberger, & Peng, 2002; Kosciessa, Kloosterman, & Garrett, 2019; Looney et al., 2018). Computational studies have shown the positive relationship between small‐world network organization and complexity on coarse‐scales (Nobukawa, Nishimura, & Yamanishi, 2019; Park et al., 2019). The signal complexity at fine scales has been associated with information processing by local neuronal assemblies, whereas variability at coarse scales has been linked to large‐scale network processing (Courtiol et al., 2016; Vakorin, Lippe, & McIntosh, 2011), both of which are critical for adapting to environmental demands (Friston et al., 1997; Garrett et al., 2013) and yield insights into neural underpinnings of fluid intelligence.

In the present study, we hypothesized a positive relationship between fluid intelligence and overall rsEEG complexity, especially in the FPN and its interactions. Since neural complexity is thought to reflect richness of brain signal (e.g., Garrett et al., 2013; Tononi et al., 1994) or its level of integrity (McIntosh et al., 2014; Sporns, Tononi, & Edelman, 2000), it is reasonable to believe that higher *gf* is associated with greater overall EEG signal complexity (Friston, 1996), especially in the resting‐state condition when brain activity is considered as a neural basis for specific tasks performance (Rasero et al., 2018). Existing evidence in that matter has yielded ambiguous outcomes. Some of them demonstrated that individuals with a high intelligence level were characterized by greater rsEEG dimensional complexity compared to those with lower IQ (Lutzenberger, Birbaumer, Flor, Rockstroh, & Elbert, 1992), whereas others did not confirm these findings (Anokhin, Lutzenberger, & Birbaumer, 1999).

In addition, we expected that the complexity at both fine‐ and coarse‐grained timescales would be related to *gf*. Previous studies indicate that keeping greater local information processing (expressed by the complexity at fine scales) along with lower long‐range interactions (represented by the coarse scales) is beneficial for cognition (McIntosh et al., 2014; Vakorin et al., 2011). The entropy at short scales might reflect the capacity of the brain network to redirect its activity which allows for better adjustment to changing environmental demands. Thus, in the present study the fine rsEEG complexity could be positively related to *gf*.

The dynamics observed at coarse‐scales refers to the achieved, relatively stable, network state (configuration) and/or the transitions between these states (the capacity to reconfigure). Hence, it could be argued that a lower level of complexity on late scales would be associated with efficient information processing, and therefore a higher level of *gf*. On the other hand, based on results linking higher fluid intelligence (*gf*) with the increased small‐world organization (Colom et al., 2006; Langer et al., 2012; Thatcher et al., 2016; van den Heuvel et al., 2009) and its association with dynamic complexity at coarse‐scales (Nakagawa, Jirsa, Spiegler, McIntosh, & Deco, 2013), this relationship may be just the opposite. Therefore, in the present study, we assumed that higher level of the coarse entropy (reflecting long‐range interactions which promote global information processing) might be related to fluid intelligence. We assumed that higher level of the coarse entropy (reflecting long‐range interactions which promote global information processing) might be related to fluid intelligence.

Furthermore, we hypothesized that there are different patterns of the relationship between the rsEEG complexity and *gf* in men and women at both fine and coarse scales. The evidence on how sex affects the relationship between rsEEG patterns and fluid intelligence is rather scarce. However, sex‐related differences in both structure and function of the brain are well documented, suggesting that men's and women's outcomes should be analyzed separately (Cahill & Aswad, 2015).

## 2 METHODS

### 2.1 Participants

The sample consisted of 119 healthy participants (57 men, mean age: 22.85 ± 2.84 years; age range: 18–30 years). There were no significant age differences between men and women (*t*[115] = .009, *p* = .99, no data on the age of two participants).

Participants were recruited via the Internet. To control for the potential effect of other individual differences (such as age, neurological and mental health history) on the relationship between mMSE features and *gf* factor, we applied restrictive inclusion criteria. From the point of view of our research question, the age factor seems to be particularly important. Previous studies (Fernández et al., 2012; Gómez, Pérez‐Macías, Poza, Fernández, & Hornero, 2013; McIntosh et al., 2014; Zappasodi, Marzetti, Olejarczyk, Tcchio, & Pizzella, 2015) have suggested that complexity measures demonstrate inverted quadratic relation with maximum between 40 and 60 years of age. For this reason only results from people aged between 18 and 30 years old were included. This age range corresponds to a period after the completion of major neurodevelopment and before the appearance of the first neurodegenerative changes.

Participants were right‐handed (verified by the Edinburgh Handedness Inventory, Oldfield, 1971) and reported no history of head trauma, psychiatric or neurological diseases. They had not taken any medications affecting the central nervous system for at least a year prior to participation in the study, and declared current nonuse of analgesic medication (self‐report). Additionally, participants were requested to avoid alcohol intake for at least 48 hr before the EEG recording, and to maintain their regular caffeine and nicotine intake for at least 24 hr (self‐report).

### 2.2 Ethical approval

The study was approved by the local ethics committees and was carried out in accordance with the ethical principles of the 1964 Declaration of Helsinki (World Medical Organization, 1996) and conformed to the ethical guidelines of the National Science Centre of Poland (2016). All participants provided written informed consent to participate in the study and were paid the equivalent of 20 euros. Participants were informed that the study concerned human cognition, and that their data would be anonymous.

### 2.3 Tests and procedure

#### 2.3.1 Fluid intelligence assessment

To assess individual level of fluid intelligence, the standard paper‐and‐pencil administered version of four tests were applied (Cattell's Culture Fair Intelligence Test, CTF‐3, Cattell & Cattell, 1973, the Paper Folding Test, Paper, Ekstrom, French, Harman, & Dermen, 1976, Number series and Pattern completion, Number and Pattern, Gągol et al., 2018). Two additional tests using computerized mode were administered (the Raven's Advanced Progressive Matrices, RAPM, Raven, Court, & Raven, 1983, and Figural Analogies, Analogies, Chuderski & Nęcka, 2012).

The CFT‐3 (Cattell & Cattell, 1973) consists of four parts (series completion, classifications, matrices, and topological relations) and includes 50 items in total (time limit: 30 min). The Paper (Ekstrom et al., 1976) requires imagining unfolding a piece of paper that has been folded and punched in several places (time limit: 10 min). Number (Gągol et al., 2018) requires finding the hidden rule according to which a sequence or an array of numbers is constructed, and completing it with the missing number (time limit: 18 min). Pattern (Gągol et al., 2018) is conceptually similar to RAPM; it consists of 16 sequences/patterns of shapes and requires choosing one shape out of a few alternatives that correctly completes each sequence or pattern of shapes (time limit: 20 min).

RAPM (Raven et al., 1983) consists of a 3 × 3 matrix figure with the lower right‐hand entry missing. Participants were asked to choose one out of eight alternatives to complete each of 36 matrices (time limit: 36 min). Analogies (Chuderski & Nęcka, 2012) consists of the 36 items, each item having an A:B::C:D format, where A, B, and C are simple patterns of shapes, B is generated from A using several geometric transformations (e.g., orientation, size, and filling), and D is an empty space to be filled with one pattern (out of four) that was transformed from C in the same way as B was transformed from A (time limit: 36 min). Three practice trials preceded each test. Both tests were computerized.

The exploratory factor analysis was used to extract the *gf* factor (explained 61.76% of total variance in the six intelligence test scores, eigenvalue = 3.71; see Table 1 for details).

**TABLE 1:** Descriptive statistics, reliabilities (Cronbach's alpha), and correlations (Pearson's *r*) for all intelligence tests were used to extract the *gf* factor (*N* = 119)

Statistics	RAPM (1)	CFT‐3 (2)	Paper (3)	Analogies (4)	Number (5)	Pattern (6)	*gf* factor
$\bar{x}$	21.03	29.02	10.45	21.76	10.72	9.42	0.00
SD	4.18	4.92	3.31	3.99	3.22	2.83	1.00
Min/max	6/31	17/38	1/16	8/29	2/17	3/16	−2.61/1.82
Skew	−.68	−.152	−.454	−.363	−.311	.036	−.284
Kurtosis	.909	−.694	−.306	.014	−.344	−.597	−.490
Alpha	.83	.80	.81	.77	.74	.74	N/A
*r*(*gf*)	.809***	.746***	.820***	.766***	.729***	.839***	
*r*(6)	.648***	.570***	.611***	.553***	.545***		
*r*(5)	.437***	.460***	.527***	.504***			
*r*(4)	.544***	.481***	.540***				
*r*(3)	.646***	.517***					
*r*(2)	.509***						

Abbreviations: CFT‐3, Cattell's Culture Fair Intelligence Test; *gf*, fluid intelligence; RAPM, Raven's advanced progressive matrices; SD, standard deviation; $\bar{x}$, mean.

*** *p* < .001.

No sex‐related difference were found for the *gf* factor (women (W): *x̄ =* − .15 ± .94, men (M): *x̄ =* .16 ± .05, *t*(117) = 1.73, *p* = .09), CFT‐3 (W: *x̄ =* 29.05 ± 5.30, M: *x̄ =* 28.98 ± 4.53, *t*(117) = −.07, *p* = .94), Analogies (W: *x̄ =* 21.42 ± 3.82, M: *x̄ =* 22.12 ± 4.18, *t*(117) = .96, *p* = .34), Paper (W: *x̄ =* 9.94 ± 2.95, M: *x̄ =* 11.00 ± 3.61, *t*(117) = 1.77, *p* = .08), and RAPM (W: *x̄ =* 20.77 ± 4.17, M: *x̄ =* 21.30 ± 4.21, *t*(117) = .68, *p* = .50). Only Number (W: *x̄ =* 10.02 ± 3.27, M: *x̄ =* 11.49 ± 3.00, *t*(117) = 2.56, *p* = .01) and Pattern (W: *x̄ =* 8.87 ± 2.65, M: *x̄ =* 10.02 ± 2.91, *t*(117) = 2.25, *p* = .03) showed significant differences. Men scored higher than women in both the Number series and the Pattern test.

Database of intelligence test results is at this URL: http://fizyka.umk.pl/~tpiotrowski/complexity/UJ_gf.csv.

#### 2.3.2 Procedure

The participants were screened using paper‐and‐pencil *gf* tests (CFT‐3, Paper, Pattern, and Number) from one to 7 days before the EEG session. During mounting the EEG cap (around 30 min), the participants read printed instructions for RAPM and Analogies as well as received several example items to solve. The EEG session started with 360 s of continuous recording during resting state with eyes open (the participants were asked to keep their eyes fixated on a cross presented at the center of the computer screen), with another 300 s recorded while the participants were required to concentrate on their respiration (not analyzed further). Then either RAPM or Analogies (the order of tests was random) were presented. The order of each test's items was fully randomized to separate the effects of difficulty from the effects of learning/fatigue and signal deterioration. After completing each set of 36 items, the EEG signal during another 240 s of resting state with eyes open was recorded (not analyzed further). This procedure was repeated for the remaining test, followed by another 240 s of the resting state recording. In addition, participants completed other cognitive tasks and personality questionnaires, which were not included in the present study. The entire EEG session lasted about 2 hr. To provide the same test conditions, each EEG examination was performed in a dimly lit room with constant artificial, independent of weather conditions. The research assistants corrected the placement of the EEG cap on the skull when necessary.

#### 2.3.3 EEG data acquisition and preprocessing

Continuous EEG was recorded at 256 Hz from 64 Ag/AgCl scalp electrodes using the Biosemi Active Two system. The electrodes were secured in an elastic cap using the 10–20 international electrode placement system, and referenced online to the common mode sense electrode located at the C1 electrode. The horizontal and vertical eye movements were monitored using four additional electrodes placed above and below the right eye, and in the external canthi of both eyes.

The acquired data were processed using MATLAB (ver. R2017a, Mathworks Inc., Natick MA) and the EEGLAB toolbox (ver. 14, Delorme & Makeig, 2004). EEG signals were downsampled to 256 Hz and high pass (>1 Hz) filtered. Bad channels were removed using an automated procedure (POP_REJCHANSPEC) based on signal *SD* (rejection threshold of >5 *SD* was used for the frequency range of 0–5 Hz and >2.5 *SD* for the frequency range 5–40 Hz). Epochs containing unusually high amplitudes were identified and removed using a threshold of 444 μV. The remaining signal was low‐pass filtered (<40 Hz) and re‐referenced to the average (common) reference. Epochs containing unusually high amplitudes were identified and removed using a threshold of 222 μV. Independent components were identified and rejected in an automated manner using the ADJUST tool (an EEGLAB plugin). The previously removed or missing channels were interpolated (POP_INTERP) purely for the sake of fitting the preprocessed data into the EEGLAB format for subsequent analysis and have not been used otherwise. Finally, we identified and removed epochs containing |amplitudes| > 111 μV. For further analysis, a number of continuous, uncut, disjoint and 10,240 samples (40 s) long epochs from each dataset were extracted.

**FIGURE 1:**
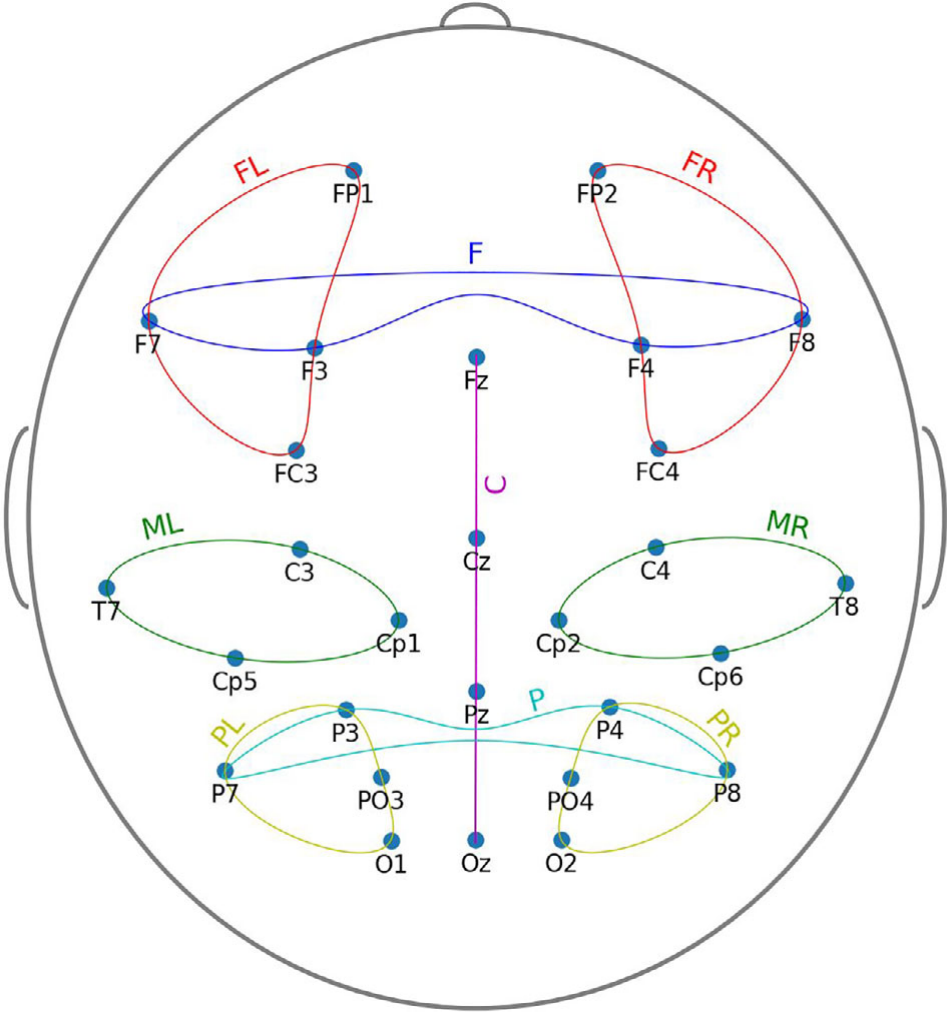
Channels and channel sets locations

The following channel sets were located in the frontal (Figure 1) (*F*: F7, F8, F3, F4), frontal left (*FL*: FP1, F7, F3, FC3), frontal right (*FR*: FP2, F8, F4, FC4), central (midline) (*C*: Fz, Cz, Pz, Oz), parietal (*P*: P3, P4, P7, P8), parietal left (*PL*: P7, P3, O1, PO3), and parietal right (*PR*: P8, P4, O2, PO4), middle left (*ML*: T7, C3, Cp5, Cp1), middle right (*MR*: T8, C4, Cp6, Cp2), regions of the scalp were selected for further analysis (Figure 1).

**FIGURE 2:**
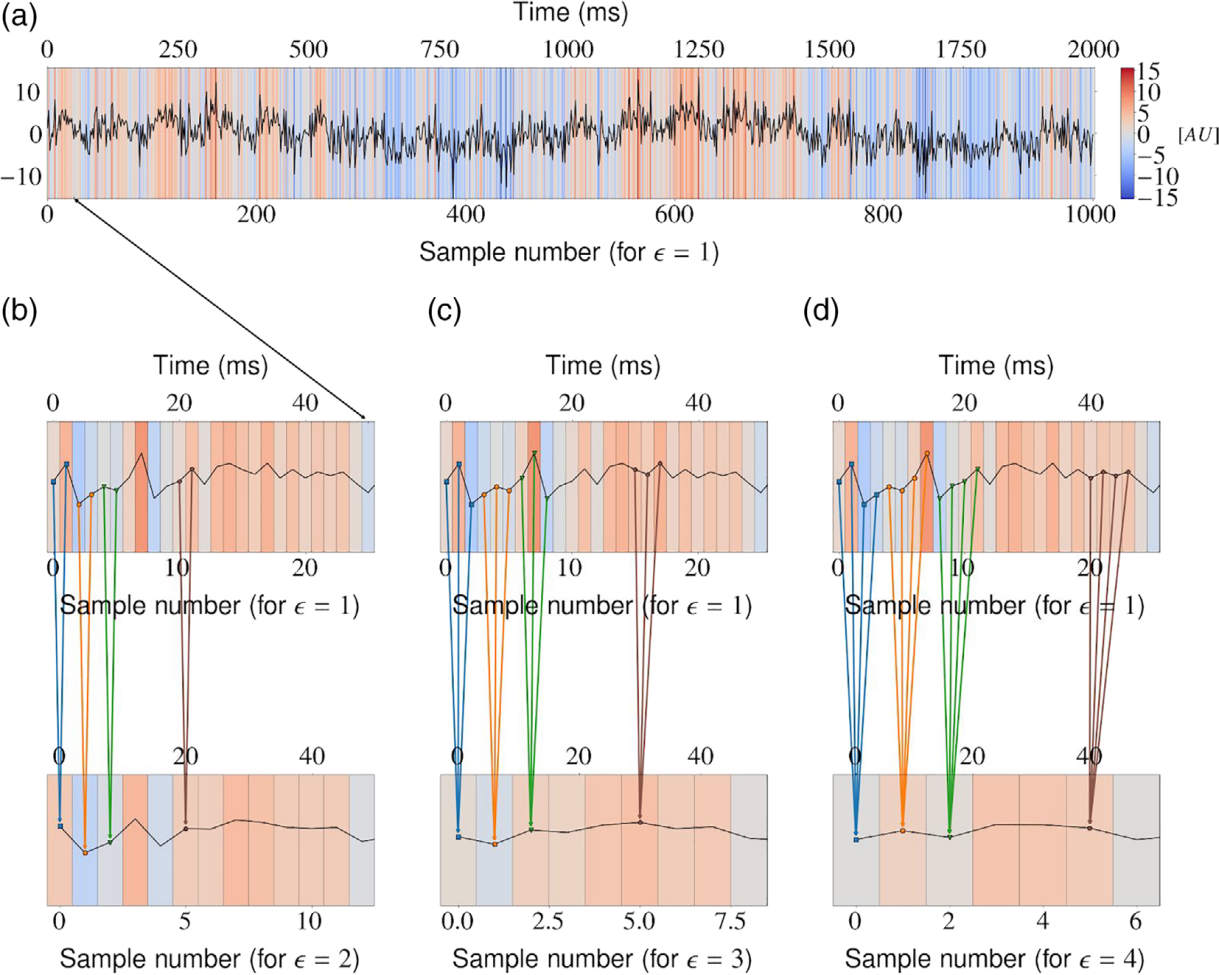
Coarse‐graining procedure was performed for each of the *p* time series considered. (a) Original *i*‐th time series, where *i =* 1,2,…,*p*. For clarity of presentation, we select the first 50 samples to be considered in the subsequent panels (b–d). Panels (b–d): The original signal is coarse‐grained (averaged) from consecutive samples over segments of increasing length (scale factor) *ε =* 2, *ε =* 3, and *ε =* 4. This process acts as a low‐pass filter and results in visible smoothing of the original signal. Note that the resulting signal is of length *N/ε*, where *N* is the length of the original signal

The preprocessing script used in this study can be found at https://github.com/IS-UMK/complexity/tree/master/Preprocessing

### 2.4 Multivariate multiscale sample entropy analysis of rsEEG

**FIGURE 3:**
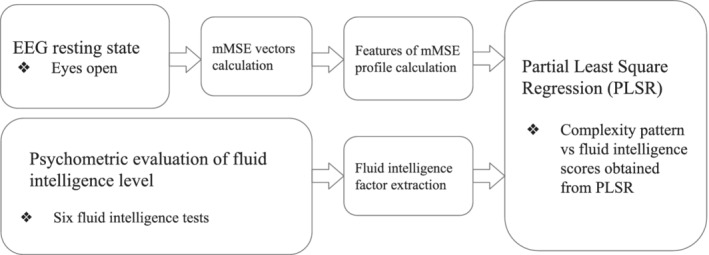
Workflow diagram

**FIGURE 4:**
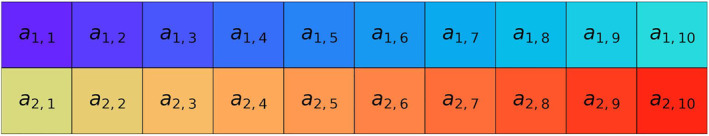
Two‐time series (*p* = 2) with *N =* 10 samples. The time series considered represent two signals for the scale factor *ε* = 1

**FIGURE 5:**
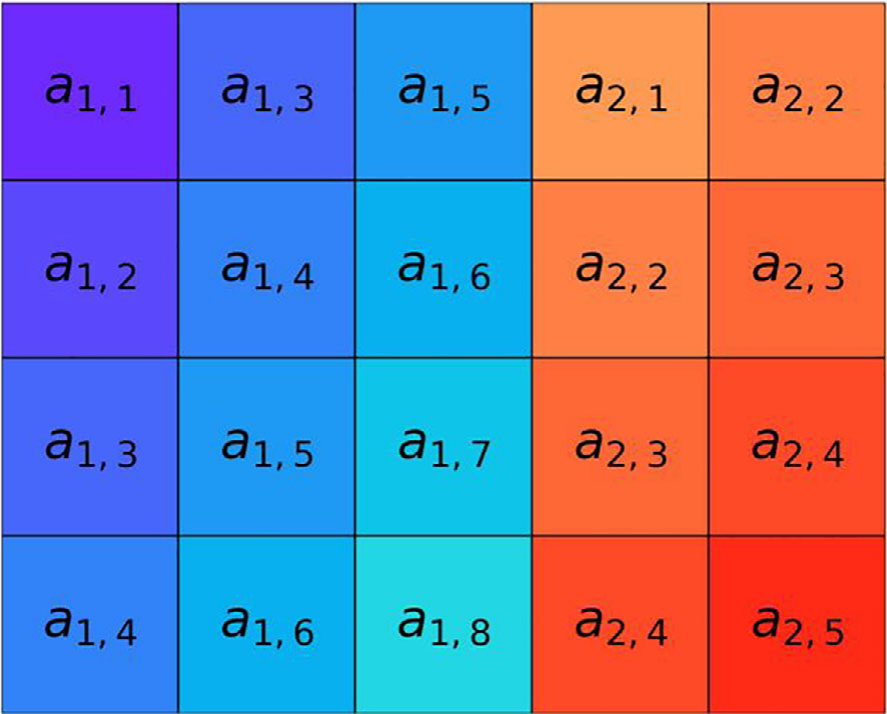
Construction of composite delay vectors (rows of the matrix) from the original *p* = 2 time series introduced in Figure 4 with *N =* 10 samples. The *i*‐th composite delay vector is obtained in this example for the embedding vector *M =* [3,2] (embedding the first signal in the 3‐dimensional space, and the second signal in the 2‐dimensional space) and the time lag (sample skipping) vector ***τ*** *=* [2,1]. The total number of composite delay vectors obtained in this way is *N–n*, where *n‐*max{*M*} x max{***τ***}. In this example *N–n* = 10*–*3 * 2 = 4. The length of each of the composite delay vectors is *m =* $\sum_{k=1}^{p}m_{k}$, where *m*_*k*_ is the *k*‐th coefficient of the embedding vector. In our case, *m* = 3 + 2 = 5

**FIGURE 6:**
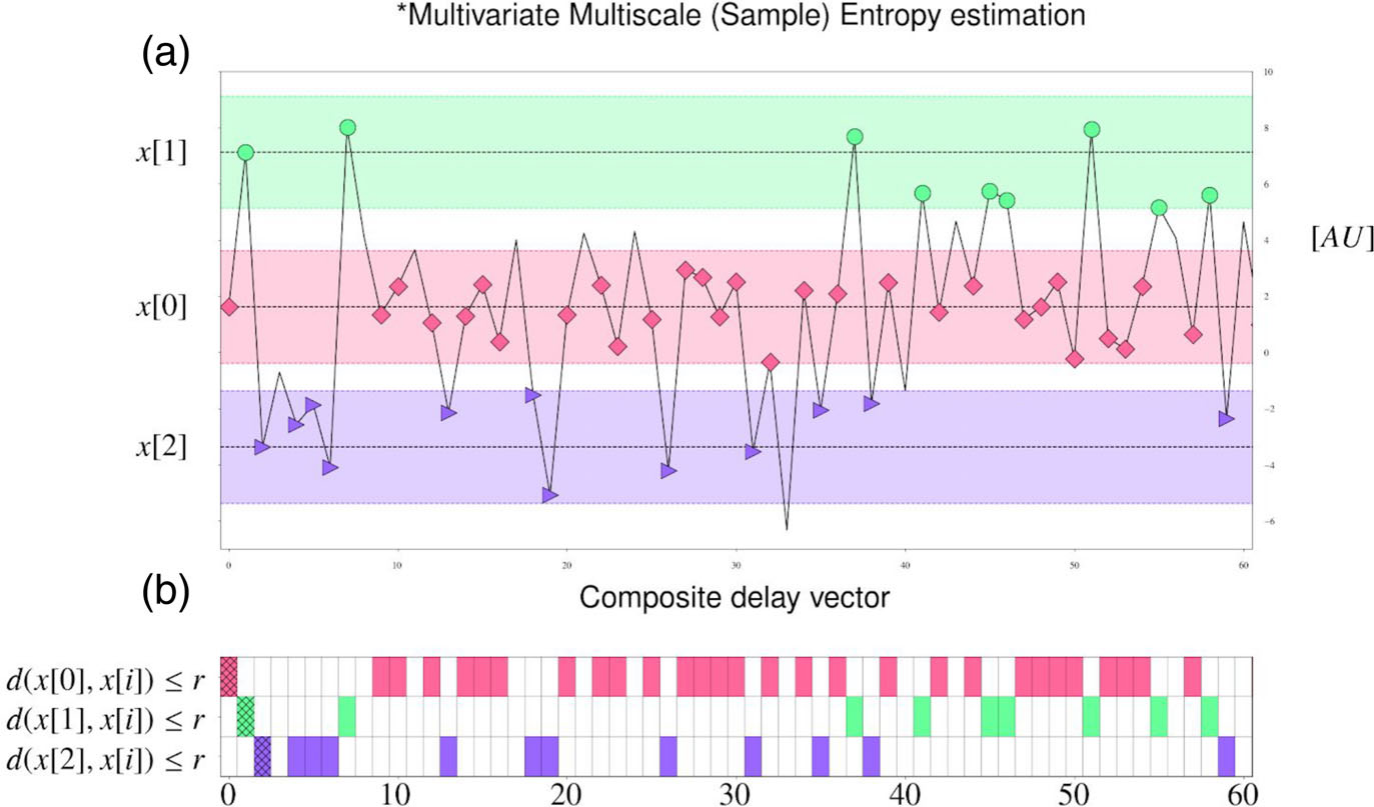
For a given similarity threshold *r >* 0, the number of composite delay vectors similar to the *i*‐th one is counted (excluding self‐matches), for *i =* 1,2,…,*N–n*. Panel (a): For *r* = 2 and for the first three composite delay vectors *x*[0], *x*[1], and *x*[2], their neighborhoods of radius *r* (pink, green, and purple stripes, respectively) are introduced. Then, all composite delay vectors lying within these neighborhoods are marked using pink diamonds, green circles, and purple triangles, respectively. Panel (b): The number of similar composite delay vectors, represented as pink, blue, and purple tiles is counted for *x*[0], *x*[1], and *x*[2], respectively. The self‐matches are excluded and are marked by the hatched area at the corresponding entry

**FIGURE 7:**
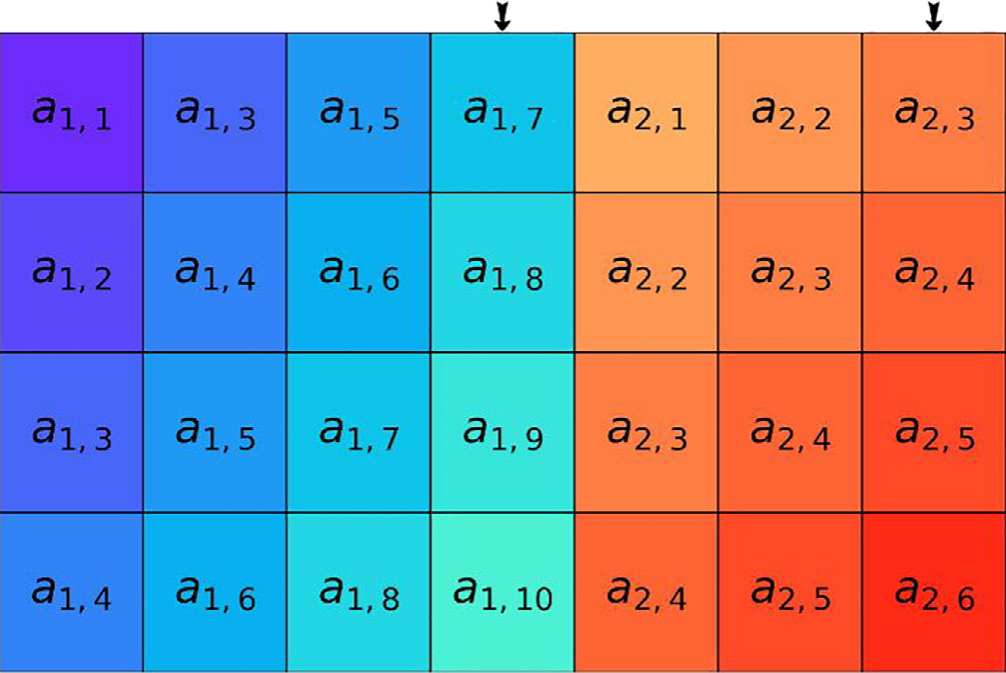
Construction of extended composite delay vectors obtained from those presented as rows of the matrix in Figure 5 by adding the next sample, simultaneously for all time series. The samples are selected according to the coefficients of the embedding vector *M =* [3,2] and the time lag vector ***τ*** *=* [2,1]. It should be noted that this procedure increases the length of each of the composite delay vectors by *p*—the number of time series considered. In the presented example, *p* = 2

Multivariate multiscale sample entropy (mMSE) analysis of rsEEG was performed using the method proposed by Looney et al. (2018). The mMSE is a multivariate extension of the MSE method based on computing the sample entropy parameter (Richman & Moorman, 2000) for coarse‐grained (averaged) time series proposed by Costa et al. (2002). We first illustrate all steps of the mMSE algorithm in Figures 2, 4, 5, 6, 7 using sample parameter values selected to maximize the clarity of the presentation of the algorithm (see also Analysis flowchart, Figure 3). Afterward, the specific choices of the mMSE parameter values used in this work are discussed and the reader is directed to the repository of the implementation of mMSE. It is worth noting that similar graphical representations of sample entropy and MSE algorithms were given by Costa et al. (2002); Grundy, Anderson, and Bialystok (2017); and Heisz, Shedden, and McIntosh (2012). To the best of the authors' knowledge, a complete graphical illustration of the mMSE algorithm has not been previously published in the literature.

Let *P*_*i*_ denote the number of composite delay vectors similar to the *i‐*th one. We introduce the normalized version of *P*_*i*_ as ${B_{i}}^{m}\left(r\right)=\left(\frac{1}{N-n-1}\right)P_{i}$ = $\frac{\mathrm{Pi}}{N-n-1}\;$ which has values between 0 (there are no composite delay vectors similar to the *i*‐th one) and 1 (all other composite delay vectors are similar to the *i*‐th one), for a given similarity threshold *r* > 0. Then, we can introduce the similarity coefficient $B^{m}\left(r\right)=\left(\frac{1}{N-n}\right)\sum_{i=1}^{N-n}B_{i}^{m}\left(r\right),$ is the average value of *B*_*i*_^*m*^ (*r*) across composite delay vectors.

It is clear that the construction of extended composite delay vectors as shown in Figure 7 produces *N‐n* such vectors. Then, the procedure of counting similar vectors presented in Figure 6 is repeated for the extended composite delay vectors, yielding *Q*_*i*_ extended composite delay vectors similar to the *i‐*th one. Similarly as before, we introduce the normalized version of *Q*_*i*_ as ${B_{i}}^{m+1}\left(r\right)=\left(\frac{1}{N-n-1}\right)Q_{i}\;$ and the similarity coefficient $B^{m+1}\left(r\right)=\left(\frac{1}{N-n}\right)\sum_{i=1}^{N-n}B_{i}^{m+1}\left(r\right)$, compare with, paragraph below the Figure 4 legend. The superscript (*m +* 1) in both *B*_*i*_^*m* + 1^(*r*) and *B*^*m* + 1^(*r*) emphasizes that these quantities are computed now for the extended composite delay vectors of length *m +* 1. Then, for the embedding vector *M*, time lag vector ***τ***, and similarity threshold *r >* 0, the mMSE coefficient at scale *ε* is of the form mMSE (*M*,***τ***,*r*,*ε*) =$-\ln\;\left(\frac{B^{m+1}\left(r\right)}{B^{m}\left(r\right)}\right).$ The mMSE vectors (values of mMSE as a function of *ε*) are obtained using the above steps performed across the range of scales *ε*.

For a single‐variate time series Pincus and Goldberger (1994) recommended the minimum number of samples to be at least 10^*m*^ for approximate entropy (ApEn), where *m* is the embedding coefficient. The papers utilizing sample entropy (Richman & Moorman, 2000) and its extensions to the multivariate case (Ahmed et al., 2012; Ahmed & Mandic, 2011; Costa et al., 2002; Looney et al., 2018) follow a similar recommendation. In the latter case, the minimum number of samples is defined as p*10^*m*^. Regarding the embedding vector *M =* [*m*_*1*_,*m*_*2*_,…,*m*_*p*_], we set the embedding vector coefficients to *m*_*k*_ *=* 2 for *k =* 1,2,…,*p*. The time delay ***τ***_k_ was set to 1 for *k =* 1,2,…,*p*, the threshold *r* measuring similarity between data points was set to *r =* .15 with time series normalized to unit variance, and the distance measure *d* was the *maximum* distance. These settings followed the guidelines proposed by Pincus and Goldberger (1994) for the ApEn measure and adopted for the SampEn‐based measures (Ahmed & Mandic, 2011; Costa et al., 2002; Looney et al., 2018; Richman & Moorman, 2000), as they ensure stability of conditional probability estimates while preserving detailed system information. Our implementation is based on modification of MATLAB scripts downloaded from:

http://www.commsp.ee.ic.ac.uk/~mandic/research/Multivariate_Complexity_Stuff/Matlab_Multivariate_Multiscale_Entropy.zip.

Our scripts are freely available based on GNU General Public License from the GitHub repository https://github.com/IS-UMK/complexity/tree/master/MMSE_vectors.

The EEG dataset used in this study to compute mMSE vectors can be found at http://fizyka.umk.pl/~tpiotrowski/complexity/UJ.mat.

### 2.5 Features of mMSE profiles

#### 2.5.1 Skewed inverted‐U shape of mMSE vectors

**FIGURE 8:**
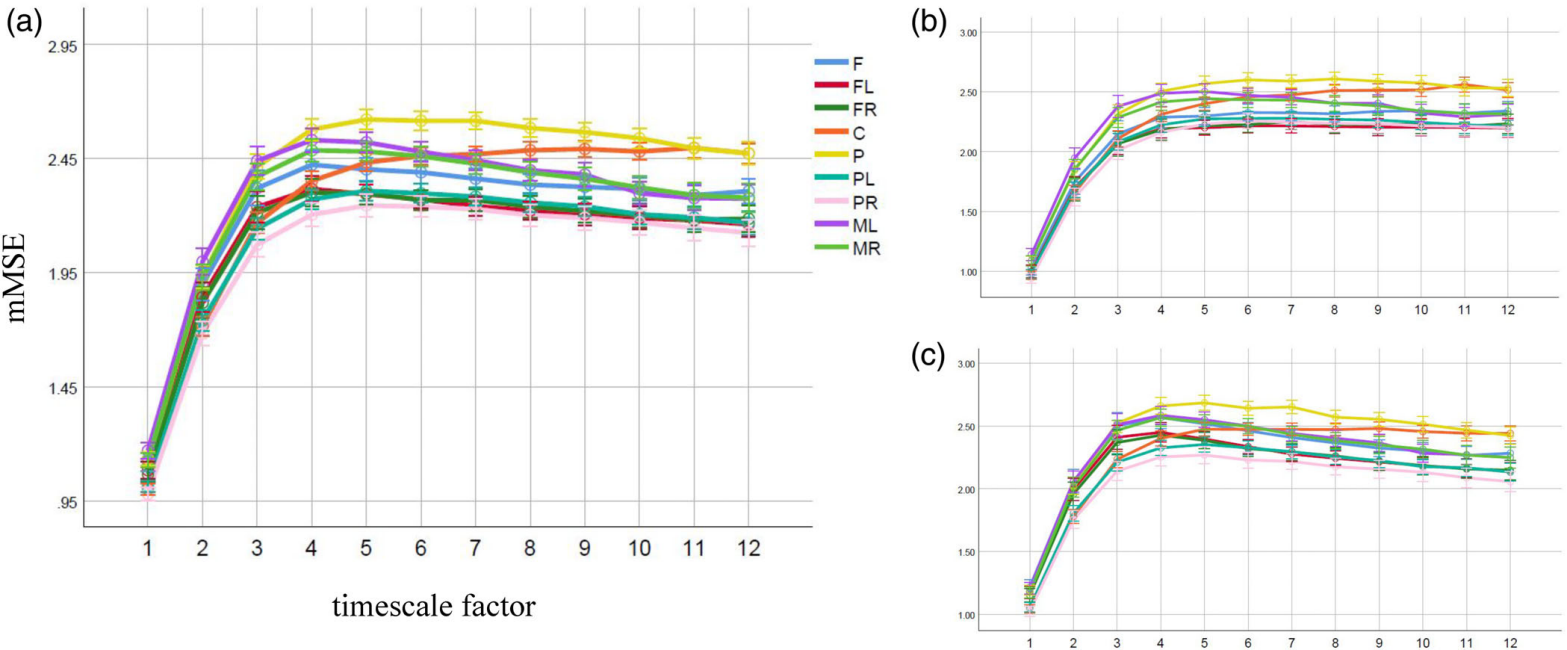
Skewed inverted‐U shapes of the mMSE vectors for each channel set in (a) the overall sample, (b) men, and (c) women. Note. The *X*‐axis represents timescales and the *Y*‐axis represents the average of the mMSE values across the subjects. Error bars represent the confidence intervals (95% CI). mMSE vectors were calculated using the following parameters for all channel sets: *m* = 2, *r* = .15, *p* = 4, *ε* = 12, where *m* is the embedding coefficient, *r* is the similarity threshold, *p* is the number of channels in a given channel set, and *ε* is the time scale factor. C, central; F, frontal; FL, frontal left; FR, frontal right; ML, middle left; MR, middle right; P, parietal; PL, parietal left; PR, parietal right; (details see Figure 1)

We obtained the mMSE vectors for each subject for frontal (F), frontal left (FL), frontal right (FR), central (C), parietal (P), parietal left (PL), parietal right (PR) and middle left (ML), middle right (MR) channel sets (Figure 1) separately, using the procedure outlined in Section 2.4 (see also Figure 3). In all of these cases, the mMSE vectors were stable and characterized by a skewed inverted‐U shape across time scales, which is typical for EEG and MEG signals (Costa et al., 2005; Courtiol et al., 2016; Grandy, Garrett, Schmiedek, & Werkle‐Bergner, 2016; Kosciessa et al., 2019; Kuntzelman, Jack Rhodes, Harrington, & Miskovic, 2018; see Figure 8). Indeed, this pattern also persists in other modalities, such as fMRI (e.g., Grandy et al., 2016; McDonough & Nashiro, 2014; McDonough & Siegel, 2018; Omidvarnia, Zalesky, Ville, Jackson, & Pedersen, 2019) or in simulation studies (e.g., Courtiol et al., 2016; Grandy et al., 2016; Kuntzelman et al., 2018). For our dataset the mMSE values stabilized at the coarse‐grained time series for scale *ε =* 12.

#### 2.5.2 *AUC*, *MaxSlope*, and *AvgEnt* features of mMSE profiles

We considered the following three features derived from the mMSE vectors, calculated for a given subject frontal (F), frontal left (FL), frontal right (FR), central (C), parietal (P), parietal left (PL), parietal right (PR) and middle left (ML), and middle right (MR) channel sets (introduced in Section 2.3.2, Figure 1).

- *Area under curve* (*AUC*), obtained by the trapezoidal approximation of the area delimited by the mMSE vector. The *AUC* feature may be viewed as the total complexity of the EEG signal represented by the mMSE vector.
- *MaxSlope*, defined as the maximum pairwise difference between the first four elements (1:4 timescales) of the mMSE vector divided by indices' difference. The *MaxSlope* feature may be viewed as representing the maximum complexity change of the EEG signal at high‐frequency fine‐scales.
- *AvgEnt*, defined as the average value of the last four elements (9:12 timescales) of the mMSE vector; the *AvgEnt* feature may be viewed as representing the baseline value of entropy of the EEG signal at low‐frequency coarse‐scales.

The scripts calculating the above features can be found at https://github.com/IS-UMK/complexity/tree/master/MMSE_features.

#### 2.5.3 Diversity of spatiotemporal complexity patterns between the channel sets

The above features characterize the mMSE vector obtained from the EEG signal from a certain, single set of channels. However, considering the purpose of our research it is important to quantify the relationship between the mMSE vectors obtained from the EEG signals from the two sets of channels for a given subject. To this end, we introduced the *difference* between two channel sets for particular mMSE features (Difference for *AUC*, *MaxSlope*, and *AvgEnt* between the following pairs of channel sets: F‐P, FL‐PL, FR‐PR, FL‐FR, PL‐PR, and ML‐MR).

### 2.6 Statistical analyses

Partial least square regression (PLSR) analysis was used to determine the extent to which the rsEEG complexity patterns were associated with *gf* factor in the overall sample, and separately in men and women samples.

In addition, we performed a series of the mix ANOVA to check the effect of sex, channel set, and brain lateralization on the mMSE features (Section S1).

#### 2.6.1 Partial least square regression analysis

The PLSR is a multivariate regression technique (Wold, Sjöström, & Eriksson, 2001) which has been designed to provide robust regression in situations where there are many correlated predictor variables and a limited number of samples, as is typical in neuroscience (Krishnan, Williams, McIntosh, & Abdi, 2011). The PLSR method decomposes the matrix of values of the predictor variables (features) **X** into orthogonal scores **T** and loadings **P** as **X = TP** in such a way as to incorporate information on both **X** and the vector representing the dependent variable **y** in **T** and **P**. More precisely, the algorithms used to find such a decomposition aim to determine scores **T** and loadings **P** in such a way as to describe as much as possible of the covariance between **X** and **y**.

In order to determine the optimal number of PLSR components (latent variables, LVs, obtained by deflating iteratively the crossproduct matrix **S = X** ^**T**^ **Y** using singular value decomposition [SVD] in the order of decreased covariance between **X** and **Y**), we used two criteria:

- the first one is based on the cross‐validated (CV) PLSR model, where the CV curve is obtained as a function of the number of components. This approach is based on the randomization test method (in this case, number of permutations: *N* = 10,000, *α* = .05 level, Van der Voet, 1994; PLS R package, Mevik, Wehrens, Liland, & Hiemstra, 2019),
- the second one simply selects the number of LVs based on the first local minimum of the CV curve as a function of the number of components (Mevik et al., 2019).

We note that the above procedure results in a single hypothesis to be verified, represented by the selected multivariate linear regression model: whether there exists or not a linear combination of independent variables matching values of a dependent variable. Thus, multiple comparisons are not performed. We also note that the PLSR does not assume that the regression residuals are normally distributed. Consequently, the assumption for the standard *t*‐test of the significance of regression coefficients is not met. To circumvent this difficulty, we have used bootstrapped estimation of confidence intervals (95% CI, number of bootstrap repetitions: *N* = 10,000) for regression coefficients (MVDALAB package, Afanador, Tran, Blanchet, & Baumgartner, 2017) to verify which independent variables were relevant to the test of our hypothesis.

The independent variables were centered and standardized.

The R script implementing the above analysis is freely available based on GNU General Public License from our GitHub repository https://github.com/IS-UMK/complexity/tree/master/PLSR.

This script uses the mMSE features and *gf* scores downloadable from http://fizyka.umk.pl/~tpiotrowski/complexity/UJ_gf_complexity.csv.

## 3 RESULTS

### 3.1 Relation of rsEEG complexity to *gf*: PLSR Analysis

**FIGURE 9:**
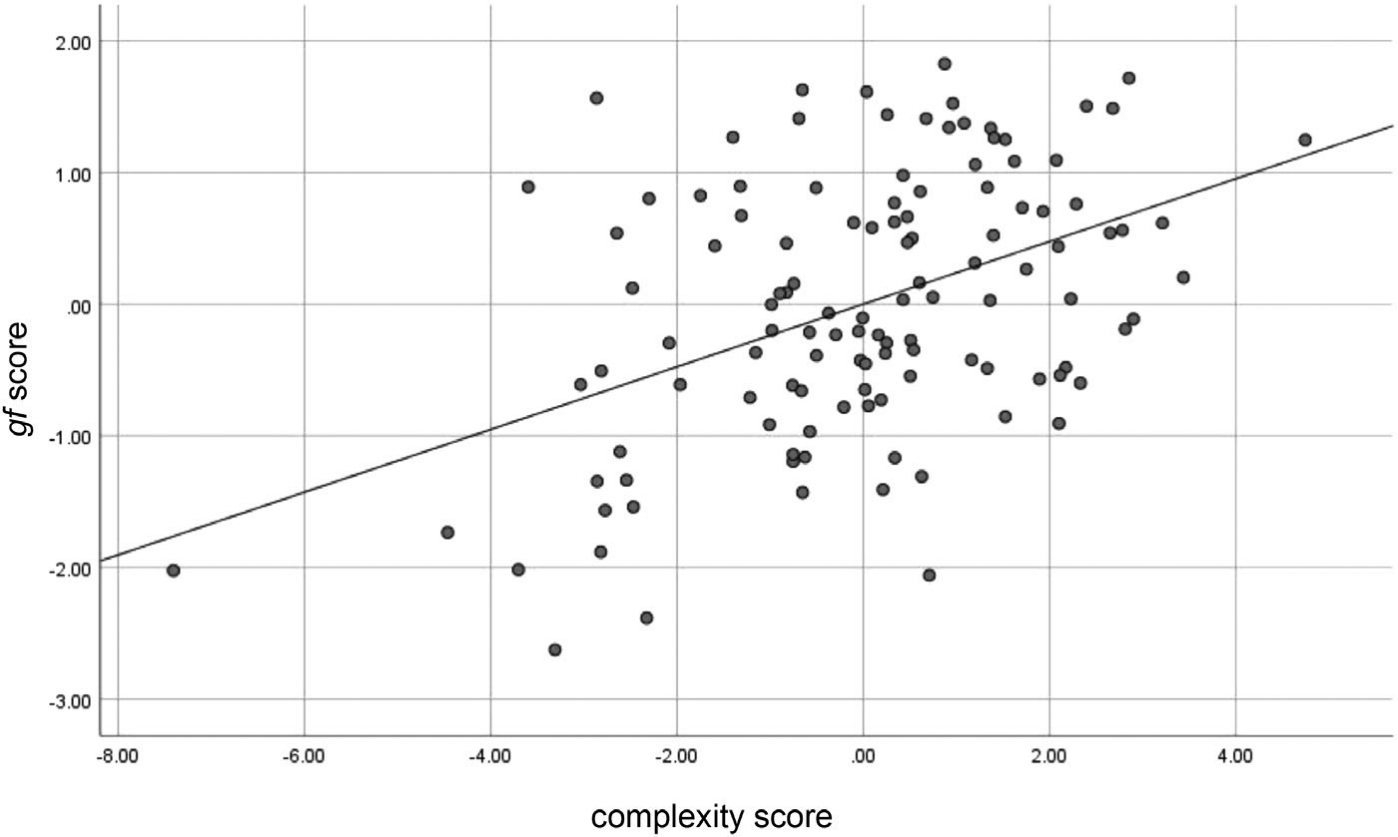
Individual subjects' complexity score versus *gf* score in the entire sample (*N* = 119)

For the overall sample (*N* = 119), the first local minimum of the CV curve is obtained for three LVs. Each of them explained respectively 19, 10, or 3% (in total 32%, Figure 9) of the shared covariance between *gf* and the complexity measures. The regression coefficients, bias‐corrected 95% bootstrapped confidence intervals (95% CI) and the bootstrap standard error (SE) for the variables relevant to the rsEEG complexity associated with *gf* are shown in Table 2. The predictors, listed in Table 2, are ordered from the most to the least strongly related to the rsEEG pattern complexity relevant to higher *gf* in groups separated into positive and negative predictors. Detailed information about the obtained regression coefficients, 95% CI and SE for all variables are provided in Table S1.

**TABLE 2:** rsEEG complexity pattern (mMSE features) relevant to *gf* obtained from PLSR analysis in the entire sample (*N* = 119)

	Predictor	Regression coefficient	95% CI	*SE*
Positive	*AvgEnt*_FL‐FR_	.133	[.053; .196]	.04
	*AvgEnt*_FL_	.120	[.058; .179]	.03
	*AvgEnt*_P_	.113	[.030; .186]	.04
	*MaxSlope*_PL‐PR_	.101	[.022; .178]	.04
	*MaxSlope*_FR‐PR_	.083	[.011; .153]	.04
	*AvgEnt*_FL‐PL_	.082	[.019; .151]	.03
	*AUC*_FL_	.076	[.019; .129]	.03
	*AUC*_FL‐PL_	.070	[.017; .123]	.03
	*AUC*_FR‐PR_	.053	[.009; .107]	.02
Negative	*MaxSlope*_PR_	−.139	[−.233; −.030]	.05
	*MaxSlope*_ML‐MR_	−.134	[−.206; −.039]	.04
	*AvgEnt*_F‐P_	−.134	[−.209; −.050]	.04
	*MaxSlope*_ML_	−.102	[−.177; −.016]	.04
	*AUC*_F‐P_	−.084	[−.157; −.007]	.04

Abbreviations: 95% CI, bias‐corrected 95% bootstrapped confidence intervals; C, central; F, frontal, FL, frontal left, FR, frontal right; ML, middle left; MR, middle right; P, parietal; PL, parietal left; PR, parietal right; SE, estimate of bootstrap standard error.

**FIGURE 10:**
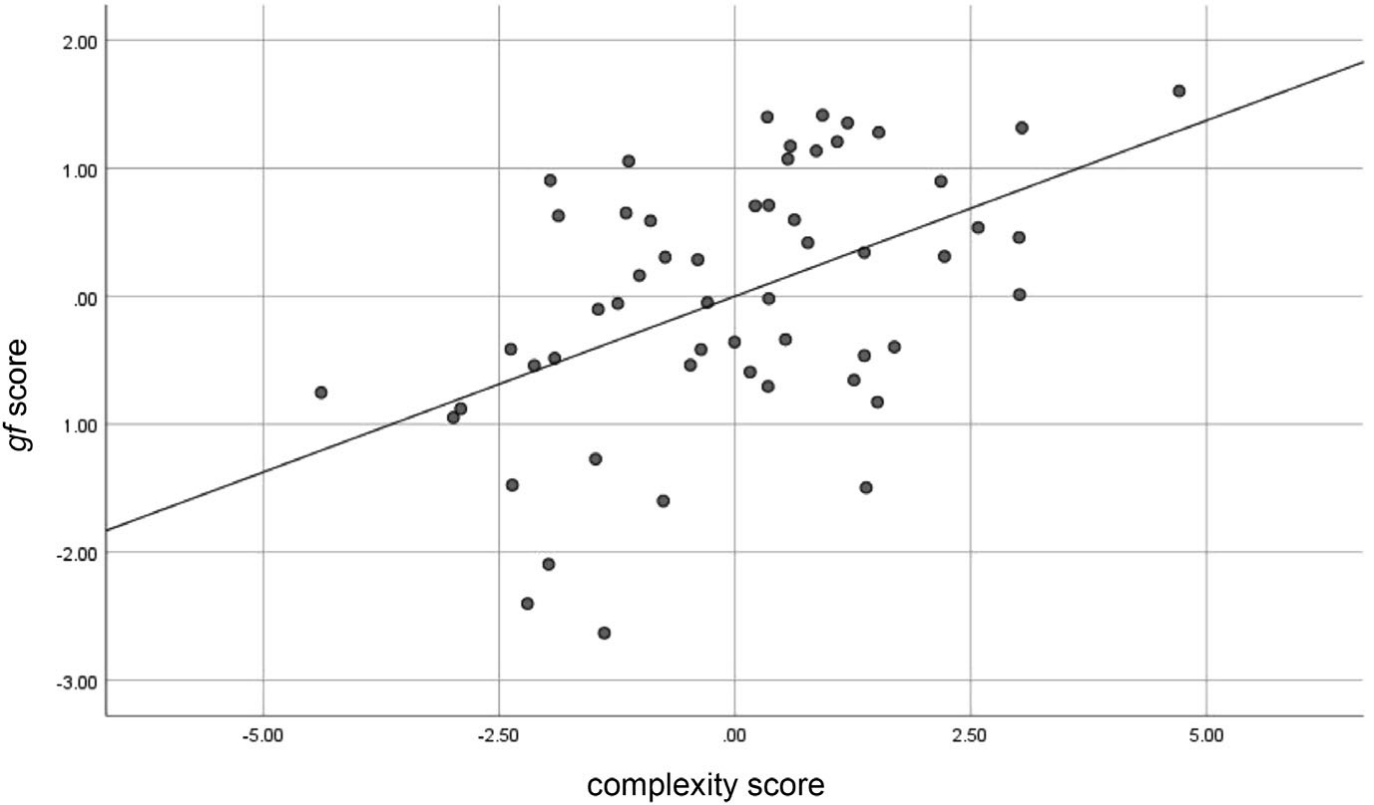
Individual subjects' complexity score versus *gf* score in men (*N* = 55)

When the PLSR analysis was conducted only in men (*N* = 55, two outliers were removed), the first local minimum allowed to extract eight LVs. This model explained respectively 24, 19, 9, 6, 6, 2, 1, and 1% (in total 68%, Figure 10) variation of the *gf*. Relevant contributors in LVs pattern significant for higher *gf* are listed in Table 3 (details, Table S2).

**TABLE 3:** rsEEG complexity pattern (mMSE features) relevant to *gf* obtained from PLSR analysis in men (*N* = 55)

	Predictor	Regression coefficient	95% CI	*SE*
Positive	*MaxSlope*_FR‐PR_	.305	[.034; .446]	.11
	*AvgEnt*_PR_	.301	[.076; .447]	.09
	*AvgEnt*_FL_	.295	[.077; .430]	.09
	*AvgEnt*_FL‐PL_	.279	[.044; .408]	.09
	*MaxSlope*_F‐P_	.276	[.028; .429]	.10
	*AvgEnt*_FL‐FR_	.195	[.002; .379]	.10
Negative	*AUC*_F‐P_	−.362	[−.466; −.093]	.10
	*AvgEnt*_PL‐PR_	−.293	[−.426; −.054]	.09
	*AvgEnt*_F_	−.254	[−.469; −.016]	.11
	*AvgEnt*_FR‐PR_	−.196	[−.426; −.054]	.09
	*AvgEnt*_F‐P_	−.172	[−.373; −.001]	.09

Abbreviations: 95% CI, bias‐corrected 95% bootstrapped confidence intervals; C, central; F, frontal, FL, frontal left, FR, frontal right; ML, middle left; MR, middle right; P, parietal; PL, parietal left; PR, parietal right; SE, estimate of bootstrap standard error.

**FIGURE 11:**
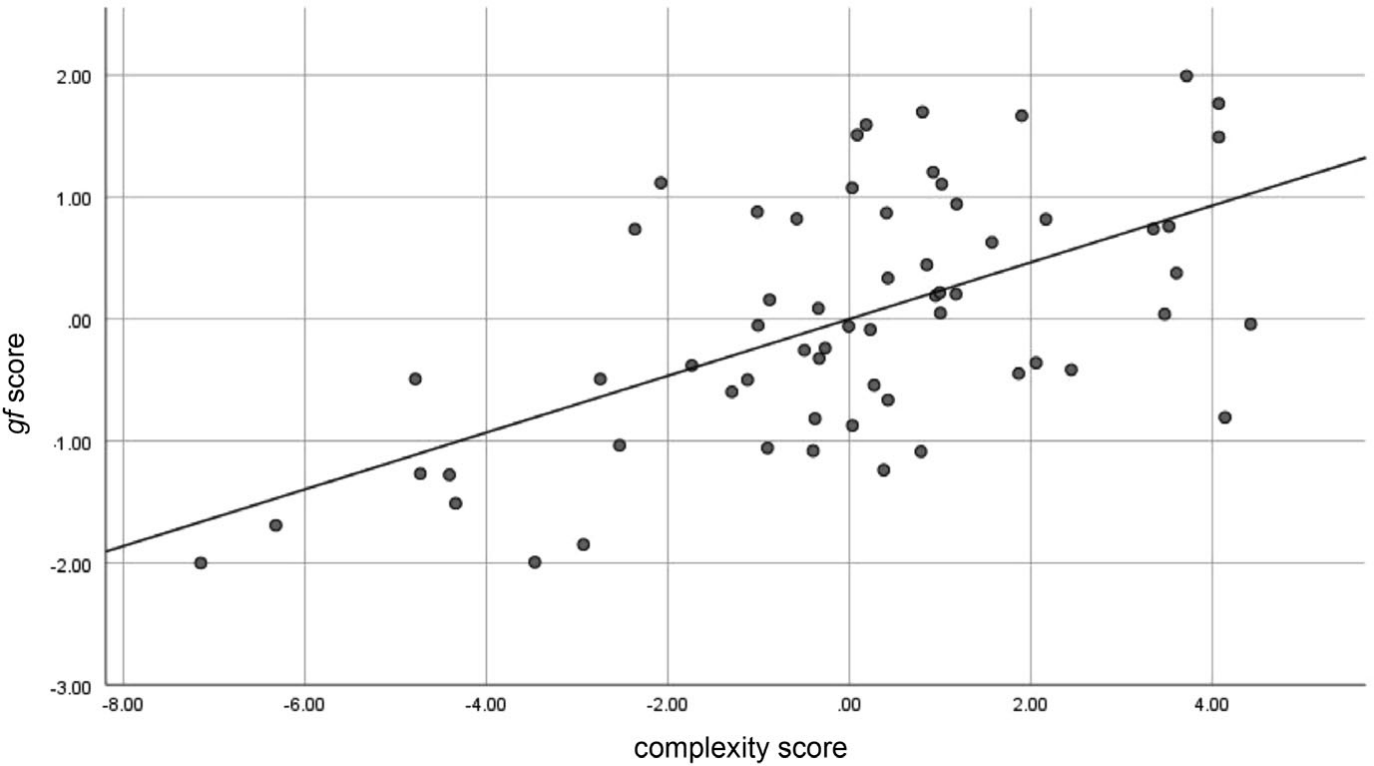
Individual subjects' complexity score versus *gf* score in women (*N* = 62)

Women (*N* = 62) demonstrated a significant relationship between the rsEEG complexity and *gf* factor (one LV, permutation test, *p* < .05, a model with one LV explained 34% variation of the *gf*, Figure 11). Significant contributors to the LV pattern relevant to *gf* are provided in Table 4. Table S3 contains the detailed results for all predictors.

**TABLE 4:** rsEEG complexity pattern (mMSE features) relevant to *gf* obtained from PLSR analysis in women (*N* = 62)

	Predictor	Regression coefficient	95% CI	SE
Positive	*AvgEnt*_FL‐FR_	.067	[.085; .032]	.02
	*AUC*_FL‐FR_	.064	[.031; .099]	.02
	*AUC*_FL‐PL_	.048	[.010; .079]	.02
	*AvgEnt*_PL‐PR_	.043	[.001; .076]	.02
	*AUC*_PL‐PR_	.042	[.001; .078]	.02
	*AUC*_FR‐PR_	.041	[.0004; .074]	.02
	*AvgEnt*_FL‐PL_	.040	[.003; .067]	.02
	*AvgEnt*_FL_	.040	[.008; .069]	.02
	*AUC*_FL_	.030	[.002; .066]	.02
Negative	*AUC*_PR_	−.064	[−.085; −.032]	.01
	*MaxSlope*_PR_	−.062	[−.087; −.029]	.01
	*MaxSlope*_ML‐MR_	−.054	[−.093; −.023]	.02
	*AvgEnt*_PR_	−.049	[−.074; −.012]	.02
	*MaxSlope*_ML_	−.049	[−.073; −.017]	.01
	*MaxSlope*_P_	−.036	[−.064; −.0003]	.02

Abbreviations: 95% CI, bias‐corrected 95% bootstrapped confidence intervals; C, central; F, frontal, FL, frontal left, FR, frontal right; ML, middle left; MR, middle right; P, parietal; PL, parietal left; PR, parietal right; SE, estimate of bootstrap standard error.

## 4 DISCUSSION

To our knowledge, this is the first study in which the multivariate extension of multiscale sample entropy (mMSE) was used to analyze spontaneous EEG data in relation to fluid intelligence (*gf*). The mMSE provides information about signal richness (complexity) in the spatiotemporal domain, that is, for different brain regions (channel sets) across different timescales. As the mMSE takes into account the cross‐correlations between variables in the time series (between electrodes), it is very useful for analyzing EEG data, typically recorded from many channels (Costa et al., 2005). Furthermore, this analysis allows us to determine not only the overall brain complexity (entropy) but also the complexity at fine‐grained (short) and coarse‐grained (long) timescales, as well as the differences between particular complexity features (Ahmed et al., 2012; Ahmed & Mandic, 2011; Looney et al., 2018). Therefore, the mMSE appears to be an excellent tool to investigate the relationship between fluid intelligence and rsEEG complexity at different timescales, which is a novel and rather unique approach in the neuroscience of individual differences.

Our findings indicate two distinct time pathways (corresponding to short and long timescales) in which fluid intelligence is related to the entropy at rest. Specifically, *gf* was mainly associated with the fronto‐parietal complexities at coarse timescales and with temporo‐parietal complexities at fine timescales (Table 2). Additionally, we found that sex influenced the relationship between fluid intelligence and spontaneous EEG complexity at short and long scales differently. This effect was observed in the absence of significant differences in *gf* test performance between men and women (Table 1).

### 4.1 *gf* and rsEEG complexity

In the current study, *gf* was associated with the overall entropy (measured by *AUC*) and the complexities at both fine and coarse timescales (expressed by *MaxSlope* or *AvgEnt*, respectively). These results were observed mainly for the FPN. However, the value of *MaxSlope* computed from signals on the electrodes located both over the frontoparietal and centro‐temporal regions was related to fluid intelligence (Table 2, Figure 9). Therefore, the present study not only reinforced the well‐documented relevance of the FPN in fluid intelligence (Dubois et al., 2018; Goh et al., 2011; Gu et al., 2015; Haier, Jung, Yeo, Head, & Alkire, 2005; Jung & Haier, 2007; Langer et al., 2012; Yoon et al., 2017) but also confirmed previous findings demonstrating that intellectual behavior is represented in the areas beyond this network (Basten, Hilger, & Fiebach, 2015; Hilger et al., 2017a; Hilger et al., 2017b; Pamplona, Santos Neto, Rosset, Rogers, & Salmon, 2015; van den Heuvel et al., 2009). However, it should be mentioned that the electrodes located over the parietal or frontal regions do not necessarily receive parietal or frontal contributions. Thus, the above conclusions should be considered with caution.

The present study revealed that better *gf* task performance was associated with greater left frontal *AUC* and *AvgEnt*, higher parietal *AvgEnt*, and larger left frontal relative to right frontal *AvgEnt* (Table 2, Figure 9). These results are partially congruent with previous evidence from resting‐state fMRI studies (McDonough & Nashiro, 2014; Omidvarnia et al., 2019; Saxe, Calderone, & Morales, 2018) demonstrating increased brain entropy in the prefrontal cortex, inferior temporal lobes and cerebellum associated with higher *gf* (Saxe et al., 2018), a positive relationship of fluid intelligence with the complexity of resting‐state networks including the FPN (Omidvarnia et al., 2019) or with the temporal variability of the middle frontal, inferior parietal and visual cortices (Yang et al., 2019). A higher intelligence level also turned out to coexist with more efficient organization of the whole‐brain network (van den Heuvel et al., 2009) or mainly the FPN (Duncan, 2010; Langer et al., 2012). Also, the global connectivity of the left lateral prefrontal cortex, both within and outside the FPN, was as a predictor of fluid intelligence (Cole et al., 2015; Cole, Yarkoni, Repovš, Anticevic, & Braver, 2012). Our results are the first to show *gf*—FPN complexity relationship similar to that demonstrated in resting fMRI studies.

We found that a larger frontal *AUC* in relation to the parietal *AUC*, determined separately for the left and right hemispheres, were positively related to *gf*. Interestingly, the difference between the frontal and parietal *AUC* (calculated for both left and right channel sets together) was negatively associated with fluid intelligence. Similar effects were observed for *AvgEnt*, except that in this case, there was a positive relationship between *gf* and the difference between the left frontal and left parietal entropies (Table 2). These results indicate that maintaining a greater within‐hemisphere advantage of frontal over parietal complexities at all timescales, and only at coarse scales along with a lower dominance of frontal over parietal entropy, computed jointly for both hemispheres, facilitates better *gf* test performance. We also cautiously suggest that both intra‐ and interhemispheric coupling in the FPN at rest are beneficial for fluid intelligence.

We found that the left frontal and parietal *AvgEnt* were positively related to *gf*. A greater advantage of left frontal *AvgEnt* over the right frontal and left parietal *AvgEnt*, was also associated with higher fluid intelligence. Considering the coarse timescales as reflecting conditions that facilitate long‐distance interactions across distributed neural assemblies (e.g., Vakorin et al., 2011), the above findings might support the role of large‐scale connections in the implementation of intellectual behaviors. A contribution of global interactions to efficient information processing remains, after all, unclear. Some authors (Thatcher et al., 2016) have claimed that the shorter rather than longer distances between brain areas are related to higher intelligence, whereas others postulated a major role of wide‐distributed areas in the brain in intellectual behaviors (Colom et al., 2006; van den Heuvel et al., 2009). Recently, both strong and weak connections have been considered responsible for the variability of intellectual behavior (Santarnecchi et al., 2017). Long‐distance connections are thought to reduce the topological distance between brain regions, which improves communication in the brain and, thereby, contributes to intellectual behavior (Bullmore & Sporns, 2009; Deco, Jirsa, et al., 2013; Deco, Ponce‐Alvarez, et al., 2013; van den Heuvel et al., 2009; Watts & Strogatz, 1998). Our outcomes are also congruent with the understanding of the role of long brain connections, proposed by Betzel and Bassett (2018 **)**, as those that provide diversity of information transmitted in the neuronal networks leading to complex brain dynamics patterns.

In light of the proposed theories concerning the functional significance of neural complexity at coarse scales (McIntosh et al., 2014), the pattern of relationship between *AvgEnt* and *gf*, determined in the current study, indicate that the increased number of long‐range interactions of the left frontal and parietal areas provides favorable conditions for better performance of fluid intelligence tasks.

We also found that *gf* was negatively associated with complexity at fine timescales (*MaxSlope*) in the right parietal and left middle regions. When *gf* was higher, there was greater right frontal and left parietal *MaxSlope* relative to *MaxSlope* in the right parietal area. Furthermore, a higher *MaxSlope* in the right middle area relative to *MaxSlope* in the left middle area was negatively related to *gf*. This pattern of the relationship between *gf* and rsEEG fine‐grained complexity suggests that intrahemispheric coupling within the FPN along with interhemispheric coupling within the middle and parietal regions might be a substrate for higher *gf*. Complexity at fine timescales has been considered to represent local information processing and within‐hemisphere functional connectivity (McIntosh et al., 2014). Recently, it has been found that greater complexity at fine timescales is related to greater integrity of white matter in the brain (McDonough & Siegel, 2018). Therefore, the results might mainly indicate that maintaining a low level of interconnections (both functional and structural) among local neuronal populations in the right parietal and middle left regions promotes *gf*.

In the present study, higher fluid intelligence was associated mainly with greater complexity at coarse timescales and lower entropy at fine timescales (Table 2). These results are consistent with recent resting‐state fMRI findings (Menon & Krishnamurthy, 2019) and highlight the usefulness of differentiating short and long scales when fluid intelligence in relation to neural complexity is examined. The pattern of relations of *gf* with *MaxSlope* and *AvgEnt* in the present study contradicts the theory implicating that information processing is the most effective when neurons desynchronize at fine timescales and synchronize at coarse timescales (Baptista & Kurths, 2008). Congruently with McDonough and Nashiro (2014), who suggested that less neural complexity is related to higher synchrony between brain areas, given outcomes indicate that better *gf* task performance coexists with synchronization at fine scales and desynchronization at coarse scales. On the contrary, the outcomes are consistent with some previous evidence implicating that decreased entropy at fine timescales and increased entropy at coarse timescales might be important features of optimized brain functioning (Farzan et al., 2017; McIntosh et al., 2014). Therefore, this study may extend the current understanding of the neural complexity at fine and coarse timescales. More studies are definitely needed to further clarify these issues.

### 4.2 *gf* and rsEEG complexity: Sex matters

We believe that this is the first study to demonstrate the different patterns of relationship between *gf* and rsEEG complexity in men and women. In this study, there were no significant sex‐related differences in fluid intelligence (Section 2.3.1, see also Table 1). This suggests that male and female brains may recruit different regions in different ways to resolve the *gf* tasks. Such an explanation has been proposed by other authors (Deary et al., 2010; Jiang et al., 2019), who found comparable results in their resting‐state fMRI studies.

By including into the data analysis the fine and coarse entropy metrics, we discovered that sex significantly affects the relation between *gf* and spontaneous EEG complexity. Specifically, in women, positive associations were found mainly with the *AUC* and *AvgEnt* in the FPN, and negative associations were observed with the parietal and left middle *MaxSlope* (Table 4). In men, fluid intelligence was predominantly related (in both directions) to the *AvgEnt* in the FPN and positively associated with the frontal relative to parietal *MaxSlope* (jointly for both hemispheres and only for right regions; Table 3).

Higher *gf* in women was associated with both greater left frontal *AUC* and *AvgEnt*, relative to the right frontal and left parietal entropies, and with lower right parietal *AUC* and *AvgEnt*. Fluid intelligence in this group was also negatively related to the parietal, especially right parietal, and left middle *MaxSlope*, as well as to the difference between the left and right middle *MaxSlope* (Table 4). These outcomes suggest that maintaining in the female brain increases of both overall complexity and complexity at coarse timescales in the left frontal area along with a decrease in these entropies in the right parietal region; and with lower entropy at fine scales in the parietal and left centro‐temporal areas, provides favorable conditions for intellectual behavior. Therefore, a high level of coarse complexity in the left anterior brain area accompanied by a low level of both entropies at coarse and fine scales in the posterior region, may be beneficial for *gf*.

In men, higher fluid intelligence was associated with greater *AvgEnt* in the left frontal and right parietal areas as well as with the bigger left frontal *AvgEnt* in relation to the right frontal and left parietal *AvgEnt* (Table 3). Furthermore, in this group, there was a negative relationship between *gf* and frontal *AvgEnt*; the difference between frontal and parietal AvgEnt (in both hemispheres and only in the right areas), as well as the difference between the left and right parietal *AvgEnt*. The favorable conditions for fluid intelligence in the male brain were provided by keeping complexity at coarse scales by increases in the left anterior and right posterior areas and decreases in the whole frontal region.

Considering the neural model that links the variability at long scales with long‐distance connections and the variability at short scales with local information processing (McIntosh et al., 2014; Vakorin et al., 2011), the pattern of the relationship between fluid intelligence and rsEEG complexity in women might indicate that increased global interconnectivity of the left frontal region and decreased parietal area accompanied by reduced connections of the parietal and left centro‐temporal regions with local neuronal populations provide favorable conditions for *gf* task performance. Consequently, in men, greater global information processing in the left frontal and right parietal regions along with reduced long‐distance connectivity of the frontal area (left and right one together) might promote intellectual behavior.

In men, interesting relations with *gf* were found in the right hemisphere: higher intelligence was associated with increased complexity at coarse scales in the parietal area and greater fine entropy in the frontal region. This effect might indicate that in the right posterior part of the brain the temporal dynamics of rsEEG signal promote global processing; whereas right anterior complexity provides favorable conditions for more local processing.

For both men and women, a high level of left frontal *AvgEnt*, also in relation to both right frontal and left parietal *AvgEnt*, and also bigger right parietal *AvgEnt*, constitute the conditions facilitating fluid intelligence (Tables 3 and 4). These relationships reflect greater lateralization of language (Tomasi & Volkow, 2012), more verbal thoughts generated during resting‐state conditions (resulting in more state transitions, Tomescu et al., 2018), or increased tendency of the brain to wander instead of settle in one state for a longer time (Chou et al., 2017). All these effects seemed to be just as likely for both sexes. In contrast to women who showed decreased right parietal *AvgEnt* associated with higher *gf*, in men, an inverse relationship was observed; that is, increased complexity at coarse scales coexisted with better *gf* task performance. These results might reflect more thoughts that involve visuospatial abilities (represented mainly in the right parietal region, e.g., Corbetta, Shulman, Francis, Miezin, & Petersen, 1995) at rest in men. In women, mostly frontal entropies were positively related to fluid intelligence, which may indicate the recruitment of prefrontal cortex during the resting state (e.g., to generate self‐reference thoughts, making future plans, etc.). Therefore, distinct patterns of relationship between *gf* and rsEEG entropy in men and women may result from different “baseline” brain activity in both sexes (see also Supporting Information results: Section S1, Figure S1).

*Gf* related to rsEEG complexity differently in men and women, may also reflect sex‐related differences in brain anatomy (Burgaleta et al., 2012; van der Linden, Dunkel, & Madison, 2017). In support of this claim, a recent paper by Shumbayawonda, Deniz Tosun, Hughes, and Abásolo (2018) revealed the relationship between gray matter integrity and MEG signal complexity only in females. Narr et al. (2007) found that cortical thickness of the frontal or temporo‐occipital regions correlated with intelligence in females and males, respectively. White matter integrity, on the other hand, turned out to be more related to *gf* in women than in men (Deary et al., 2010). Since the temporal variability of brain signals is thought to be differently associated with brain structure in men and women, it is highly recommended to include this data when investigating how intelligence and neural complexity are interrelated.

### 4.3 Limitations of the study and future directions

This study showed that sex significantly affects the relationship between resting EEG signal complexity and fluid intelligence. However, in the present study factors that might potentially account for this relationship were not controlled. Specifically, previous findings have revealed that sex hormone levels, a phase of menstrual cycle in women, or exogenous hormone administration (e.g., taking contraceptive pills) significantly affected brain functioning (Solís‐Ortiz, Ramos, Arce, Guevara, & Corsi‐Cabrera, 1994; Vogel, Beer, & Clody, 1971). Resting‐state activity is also thought to be susceptible to fluctuations of sex hormones during the menstrual cycle (Arélin et al., 2015).

Another limitation is the lack of control over mental processes that are not occupied with any particular task or external stimuli. In this case, the relationship between the rsEEG complexity and fluid intelligence may be affected by differences in spontaneous cognition (Andrews‐Hanna, Reidler, Huang, & Buckner, 2010; Christoff & Fox, 2018); for example, self‐generating thoughts, mind‐wandering, fluctuations of attention, emotional states or personality, and temperamental traits, which could be related directly to both the intelligence level and spontaneous bioelectrical activity. It appears that the relationship between EEG signal complexity and fluid intelligence may be specifically mediated by sex differences in spontaneous cognition, which has not been a subject of systematic research to date.

However, it should be noted that the last two paragraphs above describe general limitations of any resting state studies using EEG and fMRI techniques.

The biggest limitation of the present study was that there was a lack of in‐depth investigations of the *gf* factor relationship with mMSE features. Thus, the neurophysiological nature of the entropies, at fine‐grained timescales and coarse‐grained scales, remains to be elucidated. According to the theory that we often refer to in the current work (Vakorin et al., 2011), fine or coarse scales represent local or global information processing, respectively. This concept has recently met some criticism (e.g., Kosciessa et al., 2019) and, therefore, should be treated with caution. Some methodological constraints of distinguishing fine and coarse timescales have also been pointed out (Omidvarnia et al., 2019). Specifically, fine entropy was thought to be more repeatable than coarse complexity. In the present study, at the stage of analysis, when the *AvgEnt* values were determined, there was a risk that some original information from EEG was deleted and reduced into random fluctuations. Therefore, the stability of mMSE vectors should be checked with the use of internal consistency or test–retest methods, especially longitudinal stability. To date, attempts to do this have been made in a few EEG (Kuntzelman et al., 2018) and fMRI studies (McDonough & Siegel, 2018; Omidvarnia et al., 2019), and only for selected entropy algorithms.

As described in Section 2.4, mMSE analysis of rsEEG was performed in this work using the method proposed by Looney et al. (2018). As such, this approach inherits limitations of both the multiscale sample entropy (MSE) introduced by Costa et al. (2002), as well as the computation of the SampEn parameter itself (Richman & Moorman, 2000). Namely, it requires a sufficiently large number of samples for SampEn to be evaluated accurately for all time scales, which is a significant limitation, considering that the length of the available time series for scale *ε* is *N/ε*, where *N* is the length of the original signal. Indeed, this coarse‐graining procedure limited the number of time scales considered in this work to *ε*_max_ = 12. Furthermore, coarse‐graining essentially acts as a moving average low pass filter, which obfuscates the frequency domain content of the signal, as it has little ability to separate frequency bands. This fact yields analysis of the relationship between mMSE and frequency content of the EEG signal difficult (Courtiol et al., 2016), although certain progress has been made recently in this area (Kosciessa et al., 2019). On a more positive note, limitations of the previously introduced approaches to the multivariate extension of the MSE regarding the choice of extension of composite delay vectors of multivariate signals has been successfully resolved (Looney et al., 2018; see also Ahmed & Mandic, 2011).

## 5 CONCLUSIONS

In the present study, it was found that the resting EEG signal complexity, calculated with the use of mMSE features, was associated with fluid intelligence (measured by a set of tasks involving *gf*). The outcomes extend the current understanding of the relationship between *gf* and neural complexity by including into data analysis new timescales. This approach allowed us to distinguish two separate temporal paths in which different patterns of relationships between fluid intelligence and rsEEG complexity in the FPN were found. The manner in which sex affected this relationship appears to be dependent on the temporal scales.

Different patterns of these relationships were found in men and women. This finding suggests that there may be a “different baseline” brain activity (Cahill & Aswad, 2015) in both sexes, or anatomical differences between the male and female brain. *Gf* was associated with rsEEG complexity mainly in the FPN (although the effects were not restricted to this network). Therefore, including sex to the analysis of the relationship between neural complexity and intellectual behavior may allow us to better understand the nature of spontaneous fluctuations during the resting state and brain representation of fluid intelligence. While sex‐specific differences in cortical complexity in the intelligence context have been investigated, this work likely to be the first to uncover such differences in the complexity of brain dynamics.

## CONFLICT OF INTEREST

The authors declare no conflict of interest.

## DATA AVAILABILITY STATEMENT

Database of intelligence test results: https://fizyka.umk.pl/~tpiotrowski/complexity/UJ_gf.csv. EEG dataset: https://fizyka.umk.pl/~tpiotrowski/complexity/UJ.mat. Preprocessing script: https://github.com/IS-UMK/complexity/tree/master/Preprocessing. Scripts calculating mMSE vectors: https://github.com/IS-UMK/complexity/tree/master/MMSE_vectors. Scripts calculating mMSE features: https://github.com/IS-UMK/complexity/tree/master/MMSE_features. Database of gf scores and calculated mMSE features (for PLSR analysis): https://fizyka.umk.pl/~tpiotrowski/complexity/UJ_gf_complexity.csv. Script implementing PLSR analysis: https://github.com/IS-UMK/complexity/tree/master/PLSR.

## Supporting information


**Appendix** **S1**: Supporting Information

**Appendix** **S2**: Supporting Information
