# Supplementary material for: Spatiotemporal complexity patterns of resting‐state bioelectrical activity explain fluid intelligence: Sex matters
Source: Hum Brain Mapp. 2020 Aug 18;41(17):4846–65. doi: 10.1002/hbm.25162 (PMC7643359; doi:10.1002/hbm.25162)
Supplement: Supplementary file 1 — Appendix S1: Supporting Information [file HBM-41-4846-s001.pdf]

S.1. The effect of sex, channel set, and brain lateralization on the *MMSE* features

S.1.1. The effect of sex and channel set

A mixed ANOVA showed the main effects of both sex (F(1,117) = 4.31, p = .04, η^2_p = .036, power = .539) and channel set (F(6.29,736.40) = 58.42, p < .001, η^2_p = .333, power = 1.0) on *AUC*. Specifically, there was a significantly (p < .05) higher *AUC* in women (M = 24.94, SD_x = .21) than in men (M = 24.32, SD_x = .21). The greatest *AUC* (M = 26.82, SD = 2.04) was found at the parietal channel set, significantly (p < .001) higher than *AUC* at the remaining channel sets (Fig. 9, top panel - a). The lowest *AUC* was observed at frontal left (M = 23.69, SD = 2.32), frontal right (M = 23.70, SD = 2.65), parietal left (M = 23.58, SD = 2.21), and parietal right (M = 22.97, SD = 2.68) channel sets. *AUC* for the frontal left, frontal right, parietal right, and parietal left channel sets were not significantly different from each other (p > .05), whereas all other differences were significant (p < .001). Central (M = 25.34, SD = 1.87), frontal (M = 24.85, SD = 2.59), middle left (M = 25.54, SD = 2.24) and middle right (M = 25.27, SD = 2.27) channel sets were characterized by similar (p > .05) moderate general level signal complexity.

The analysis also revealed significant main effect of sex (F(1,117) = 13.48, p < .001, η^2_p = .103, power = .954) and channel set (F(5.42,634.09) = 16.71, p < .001, η^2_p = .125, power = 1.0) on *MaxSlope*. Women showed greater *MaxSlope* than men (W: M = .49, SD_x = .01 M: M = .45, SD_x = .01 , p < .001). The greater *MaxSlope* values was revealed at parietal (M = .50, SD = .08), middle left (M = .51, SD = .11) and middle right (M = .50, SD = .10) channel sets (they did not differ significantly, p > .05, all other differences were significant at p <.05 level). The lowest *MaxSlope* were found at parietal right (M = .42, SD = .07 – the lowest one) and parietal left (M = .44, SD = .07) channel sets (they did not differ significantly, p > .05). All other differences were significant at p <.01 level, with three exceptions for parietal left *MaxSlope*. *MaxSlope* at parietal left channel set did not differ significantly from frontal left (M = .47, SD

= .12), frontal right (*M* = .46, *SD* = .12) and central (*M* = .46, *SD* = .05) ($p > .05$). *MaxSlope* at frontal left and frontal right did not differ from frontal and central channel sets ($p > .05$).

There was also revealed significant main effect of channel set on *AvgEnt* ($F(6.09,712.20) = 49.78$, $p < .001$, $\eta^2_p = .298$, power = 1.0) The main effect of sex failed to reach significance level ($F(1,117) = 2.81$, $p = .096$, $\eta^2_p = .023$, power = .383). The greater *AvgEnt* values was revealed at parietal (*M* = 2.52, *SD* = .23) and also at central (*M* = 2.49, *SD* = .21) channel sets (all other differences were significant at p <.001 level). While, the lowest signal complexity were found at frontal left (*M* = 2.19, *SD* = .27), frontal right (*M* = 2.20, *SD* = .29), parietal left (*M* = 2.20, *SD* = .25) and parietal right (*M* = 2.16, *SD* = .30) channel sets (they did not differ significantly, $p > .05$, all other differences were significant at p <.005 level). Moderate level of complexity characterized middle left (*M* = 2.31, *SD* = .29), middle right (*M* = 2.32, *SD* = .29) and frontal (*M* = 2.31, *SD* = .27) channel sets.

Additionally, significant channel set × sex interaction was observed for *AUC* ($F(6.29,736.40) = 2.53$, $p = .018$, $\eta^2_p = .021$, power = .857) and *MaxSlope* ($F(5.42,634.09) = 4.10$, $p < .001$, $\eta^2_p = .034$, power = .966). For *AvgEnt* the interaction effect of channel set and sex failed to reach significance level ($F(6.09,712.20) = .40$, $p = .879$, $\eta^2_p = .003$, power = .171) (Fig. 9, bottom panel - c). In women compared to men there was a significantly higher *AUC* at frontal (W: *M* = 25.51, *SD* = 2.40, M: *M* = 24.13, *SD* = 2.55, $p < .005$), frontal left (W: *M* = 24.31, *SD* = 2.18, M: *M* = 23.02, *SD* = 2.30, $p < .005$) and frontal right (W: *M* = 24.22, *SD* = 2.42, M: *M* = 23.14, *SD* = 2.80, $p < .05$) channel sets (Fig. 9, top panel - a). and greater *MaxSlope*, at frontal (W: *M* = .52, *SD* = .15, M: *M* = .44, *SD* = .10, $p < .002$), frontal left (W: *M* = .51, *SD* = .14, M: *M* = .42, *SD* = .09, $p < .001$), frontal right (W: *M* = .49, *SD* = .13, M: *M* = .43, *SD* = .10, $p < .002$), parietal (W: *M* = .52, *SD* = .10, M: *M* = .48, *SD* = .07, p < .05) and middle right (W: *M* = .52, *SD* = 10, M: *M* = .47, *SD* = .09, $p < .05$) channel sets (Fig. 9, middle panel - b).

The Huynh-Feldt epsilon correction was either ε = .787 (*AUC*), ε = .677 (*MaxSlope*), ε = .761 (*AvgEnt*) and Bonferroni correction for *multiple comparisons*.

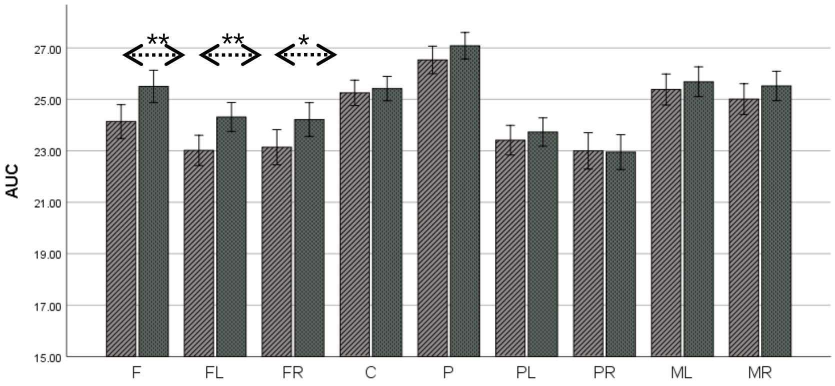

a.

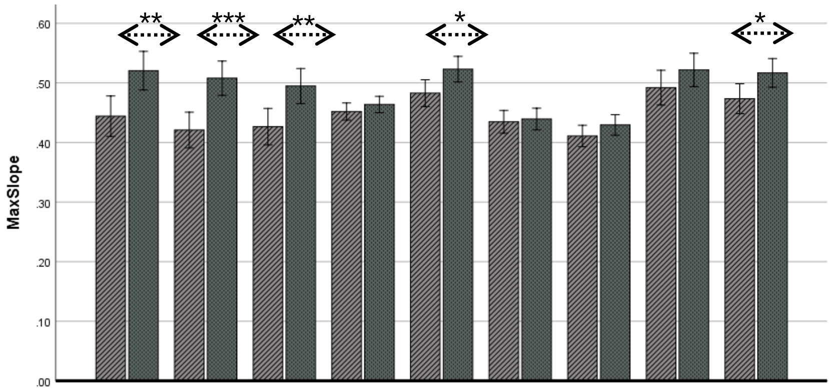

b.

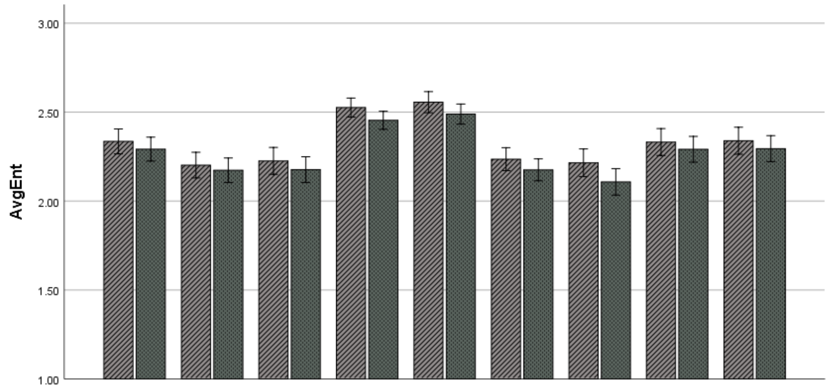

c.

Figure S1. Interaction effect of sex and channel set e on *MMSE* features

Note. Y-axis represents the average of the particular *MMSE* feature (*AUC* – panel a, *MaxSlope* – panel b, *AvgEnt* – panel c) across the subjects. Light gray bar represents the average of the particular *MMSE* feature in men, dark gray bar represents the average of the particular *MMSE* feature in women. Error bars represent CI (95%). F – frontal, FL – frontal left, FR – frontal

right, C – central, P -parietal, PL – parietal left, PR – parietal right, ML – middle left, MR – middle right. *** p < .001, ** p < .005, * p < .05.

3.1.2. The effect of brain lateralization

We performed additional mix ANOVA to test whether left hemisphere complexity features were differ from right hemisphere in men and women. We included the following channel sets in the analysis: frontal left, frontal right, parietal left, parietal right, middle left, middle right channels for *AUC*, *MaxSlope*, and *AvgEnt* features of the *MMSE*. The analysis scheme was as follows: three regions (frontal, parietal, middle) × two hemispheres × sex.

Only main effect of hemispheres (left hemisphere > right hemisphere) were significant for *AUC* ($F(1, 117) = 2.53$, $p = .018$, $\eta^2_p = .021$, power = .857, LH: $M = 24.26$, $SD_x = .16$; RH: $M = 23.97$, $SD_x = .18$) and *MaxSlope* ($F(1,117) = 4.33$, $p = .04$, $\eta^2_p = .036$, power = .541, LH: $M = .47$, $SD_x = .01$; RH: $M = .46$, $SD_x = .01$). For *AvgEnt* no effect of hemisphere reached significance.

We have not shown either significant interaction effects between the hemisphere and the region, the hemisphere and the sex, and the hemisphere, the region and the sex.
