# Supplementary material for: Spatiotemporal complexity patterns of resting‐state bioelectrical activity explain fluid intelligence: Sex matters
Source: Hum Brain Mapp. 2020 Aug 18;41(17):4846–65. doi: 10.1002/hbm.25162 (PMC7643359; doi:10.1002/hbm.25162)
Supplement: Supplementary file 2 — Appendix S2: Supporting Information [file HBM-41-4846-s002.pdf]

S.2. Complexity patterns in relations to the *gf* factor: PLSR analysis

Table S1

Complexity (*MMSE* features) pattern relevant to *gf* obtained from PLSR analysis in overall sample (N = 119), 3 LVs

variable	Actual	2.5%	97.5%	boot.mean	Skewness	Bias	SE
AUC_{F}	-0.044458588	-0.108117663	0.0180149873	-0.041936380	-0.1799129751	2.522207e-03	0.03198081
AUC_{FL}	0.075646389	0.018810518	0.1285784200	0.072833984	0.1014188680	-2.812405e-03	0.02757010
AUC_{FR}	0.009973641	-0.043939655	0.0792659749	0.014044283	0.2832774336	4.070642e-03	0.03156975
AUC_{C}	-0.030443747	-0.132023164	0.0664607023	-0.033017101	0.0001183775	-2.573354e-03	0.05018292
AUC_{P}	0.048897917	-0.033736242	0.1336820436	0.046979740	0.1249803003	-1.918177e-03	0.04215635
AUC_{PL}	-0.004884235	-0.067575405	0.0557816013	-0.006469801	0.0413529575	-1.585566e-03	0.03183562
AUC_{PR}	-0.050638180	-0.095508678	0.0001142901	-0.048638005	0.0939075500	2.000174e-03	0.02435887
AUC_{ML}	-0.034087407	-0.108785186	0.0439602079	-0.031174960	-0.0518949361	2.912448e-03	0.03879305
AUC_{MR}	0.030317496	-0.052027041	0.0993789381	0.024806544	-0.0847754404	-5.510952e-03	0.03886186
AUC_{F-P}	-0.084057794	-0.156827248	-0.0072892502	-0.079947823	-0.1169534133	4.109972e-03	0.03822534
AUC_{FL_FR}	0.071240328	-0.019693545	0.1329311764	0.062154899	-0.2850644236	-9.085428e-03	0.03879551
AUC_{PL-PR}	0.045570332	-0.012579228	0.0942635020	0.042107657	-0.0329816057	-3.462675e-03	0.02703947
AUC_{ML-MR}	-0.064224017	-0.124524120	0.0196424121	-0.055922168	0.1245579662	8.301849e-03	0.03666857
AUC_{FL-PL}	0.070091359	0.016519873	0.1229117640	0.068764923	0.0451369327	-1.326436e-03	0.02712467
AUC_{FR-PR}	0.052701817	0.008617555	0.1069704572	0.054795034	0.1792207559	2.093217e-03	0.02488329
$AvgEnt_{F}$	-0.050999752	-0.129364937	0.0204516040	-0.049326184	-0.2723894168	1.673569e-03	0.03798302
$AvgEnt_{FL}$	0.119594431	0.058498761	0.1787837359	0.115985328	0.2220926989	-3.609103e-03	0.03077424
$AvgEnt_{FR}$	-0.001256433	-0.044647258	0.0518643438	0.001633240	0.1200324405	2.889673e-03	0.02441029
$AvgEnt_{C}$	-0.018012899	-0.131985201	0.0820414299	-0.022251236	-0.1520496133	-4.238338e-03	0.05448578
$AvgEnt_{p}$	0.112545097	0.029775328	0.1863201856	0.108047462	0.0218210084	-4.497635e-03	0.03981848
$AvgEnt_{PL}$	0.026138682	-0.049567588	0.0903463993	0.022450503	-0.1460104448	-3.688179e-03	0.03555700
$AvgEnt_{PR}$	0.031892216	-0.033793466	0.1072748343	0.034016485	0.1281449483	2.124269e-03	0.03580550
$AvgEnt_{ML}$	-0.004826474	-0.090877758	0.0756229760	-0.006073360	-0.0569322975	-1.246886e-03	0.04259522
$AvgEnt_{MR}$	0.013275234	-0.068076384	0.0766685664	0.006903750	-0.1641031340	-6.371484e-03	0.03659720
$AvgEnt_{F-P}$	-0.134129062	-0.209170942	-0.0497085987	-0.128863709	-0.0736620873	5.265353e-03	0.04066527
$AvgEnt_{FL-FR}$	0.132772940	0.053173811	0.1960148994	0.125119768	-0.0350624588	-7.653172e-03	0.03624528
$AvgEnt_{PL-PR}$	-0.009760815	-0.086925577	0.0515206778	-0.014642225	-0.1618540336	-4.881409e-03	0.03548553
$AvgEnt_{ML-MR}$	-0.018931711	-0.092536436	0.0697374764	-0.013173918	0.1230082235	5.757794e-03	0.04149935
$AvgEnt_{FL-PL}$	0.082408216	0.019257527	0.1506772864	0.081911858	0.2432007478	-4.963574e-04	0.03362386
$AvgEnt_{FR-PR}$	-0.030320601	-0.086358928	0.0260848126	-0.029825591	0.0102633267	4.950099e-04	0.02850987
$MaxSlope_{F}$	0.025660699	-0.030491982	0.1003322073	0.030204646	0.2956200667	4.543947e-03	0.03347006
$MaxSlope_{FL}$	-0.017931016	-0.066631612	0.0282983297	-0.017236964	-0.1588755488	6.940520e-04	0.02445671
$MaxSlope_{FR}$	0.002672783	-0.056728451	0.0613909085	0.002712921	0.0277309216	4.013766e-05	0.02995712
$MaxSlope_{C}$	-0.018671232	-0.118832849	0.0876071313	-0.018342234	0.1644713084	3.289985e-04	0.05270997
$MaxSlope_{P}$	-0.031790614	-0.110201147	0.0557216550	-0.029227591	0.1032174073	2.563023e-03	0.04236725
$MaxSlope_{PL}$	-0.020543378	-0.116506468	0.0787821253	-0.018597287	0.0290478846	1.946092e-03	0.05070137
$MaxSlope_{PR}$	-0.138848273	-0.232990385	-0.0297582826	-0.131153611	-0.0153640049	7.694662e-03	0.05218916
$MaxSlope_{ML}$	-0.102156352	-0.177079530	-0.0161735062	-0.097016280	-0.0232412168	5.140073e-03	0.04109944
$MaxSlope_{MR}$	0.038565803	-0.075214126	0.1338256558	0.035084208	-0.2632462445	-3.481595e-03	0.05324328
$MaxSlope_{F-P}$	0.043245418	-0.030725112	0.1217842539	0.045435225	0.0260744269	2.189807e-03	0.03850447
$MaxSlope_{FL-FR}$	-0.025310720	-0.106451248	0.0501229326	-0.024697693	-0.1322159081	6.130268e-04	0.03958887
$MaxSlope_{ML-MR}$	-0.133647549	-0.205889147	-0.0387509360	-0.125965734	0.1866959602	7.681815e-03	0.04269145
$MaxSlope_{FL-PL}$	-0.005638562	-0.071050957	0.0609299814	-0.005595331	-0.0225220069	4.323109e-05	0.03357138
$MaxSlope_{FR-PR}$	0.083471802	0.010741964	0.1533476862	0.080465280	0.1171849283	-3.006522e-03	0.03644389
$MaxSlope_{PL-PR}$	0.101431439	0.022094111	0.1784352429	0.096948729	0.1873093261	-4.482710e-03	0.03979018

Table S2
Complexity (*MMSE* features) pattern relevant to *gf* obtained from PLSR analysis in men (N = 55), 8 LVs, local minmum

variable	Actual	2.5%	97.5%	boot.mean	Skewness	Bias	SE
AUC$_{F}$	-0.162994053	-0.32044097	0.0354973861	-0.147776887	0.112134844	0.015217165	0.09113638
AUC$_{FL}$	-0.008303939	-0.20924288	0.2137331778	0.001744176	-0.001882610	0.010048115	0.10785784
AUC$_{FR}$	0.044523033	-0.10964313	0.2037451464	0.058244677	-0.310998215	0.013721644	0.08014549
AUC$_{C}$	-0.153679792	-0.35037592	0.0999287972	-0.137612413	0.179716960	0.016067379	0.11632676
AUC$_{P}$	0.283429345	-0.04660017	0.4637697291	0.212687220	-0.095673311	-0.070742125	0.13016801
AUC$_{PL}$	0.194947796	-0.07803578	0.3563432760	0.143544490	-0.089460095	-0.051403306	0.11044430
AUC$_{PR}$	-0.031261464	-0.26301771	0.1488430746	-0.037538748	-0.433467100	-0.006277284	0.10420453
AUC$_{ML}$	0.031898526	-0.21221120	0.2923788682	0.045731774	-0.109961167	0.013833248	0.12951737
AUC$_{MR}$	0.099569487	-0.16788169	0.3058493500	0.076532757	-0.134439725	-0.023036730	0.12022030
AUC$_{F-P}$	-0.362080027	-0.46575625	-0.0925929848	-0.296616053	0.357242495	0.065463973	0.09520848
AUC$_{FL-FR}$	-0.067919201	-0.28274388	0.1524400180	-0.076762588	0.235537232	-0.008843387	0.11050679
AUC$_{PL-PR}$	0.166474981	-0.04113335	0.3387504546	0.134433770	0.323827554	-0.032041211	0.09755255
AUC$_{ML-MR}$	-0.072915244	-0.24933542	0.1771536543	-0.036027328	0.018370449	0.036887916	0.10924400
AUC$_{FL-PL}$	-0.163789015	-0.31591242	0.0976883469	-0.114313082	0.106407852	0.049475933	0.10598614
AUC$_{FR-PR}$	0.061941686	-0.07950615	0.2544687005	0.079774710	0.162375493	0.017833024	0.08443613
AvgEnt$_{F}$	-0.254077949	-0.46930533	-0.0164425452	-0.250346592	0.128651183	0.003731357	0.11534777
AvgEnt$_{FL}$	0.295455782	0.07718823	0.4298639604	0.252546495	0.023356295	-0.042909288	0.08898567
AvgEnt$_{FR}$	0.081537390	-0.09825209	0.1940974587	0.051537067	-0.106210659	-0.030000323	0.07373059
AvgEnt$_{C}$	-0.368264931	-0.57842661	0.0719906216	-0.267749234	0.186329803	0.100515697	0.16625163
AvgEnt$_{P}$	-0.079085862	-0.28719207	0.1518015703	-0.058868052	-0.172446275	0.020217811	0.11176952
AvgEnt$_{PL}$	-0.020688062	-0.20170895	0.1851518290	-0.012260824	0.096890477	0.008427239	0.09691455
AvgEnt$_{PR}$	0.301287234	0.07584958	0.4470458128	0.260401185	0.053254350	-0.040886049	0.09479128
AvgEnt$_{ML}$	0.022554024	-0.21895826	0.2002249298	-0.010994177	0.020761584	-0.033548201	0.10745487
AvgEnt$_{MR}$	-0.086197506	-0.33240237	0.1564106982	-0.093026782	0.108283863	-0.006829276	0.12464501
AvgEnt$_{F-P}$	-0.172323372	-0.37336695	-0.0008611312	-0.186217489	0.007737767	-0.013894117	0.09448345
AvgEnt$_{FL-FR}$	0.195427989	0.00218042	0.3794990950	0.187210000	0.117546801	-0.008217988	0.09619376
AvgEnt$_{PL-PR}$	-0.292829398	-0.42582395	-0.0543559508	-0.246004717	0.081247813	0.046824681	0.09337691
AvgEnt$_{ML-MR}$	0.115745913	-0.11559700	0.2831467902	0.088739946	-0.084290491	-0.027005967	0.10076203
AvgEnt$_{FL-PL}$	0.278547502	0.04406129	0.4076295323	0.233460375	-0.160741736	-0.045087127	0.09152284
AvgEnt$_{FR-PR}$	-0.195699018	-0.39320597	-0.0080191036	-0.188987184	-0.195671286	0.006711834	0.09785604
MaxSlope$_{F}$	0.191067246	-0.04042043	0.4111735357	0.186104133	-0.026432530	-0.004963113	0.11539105
MaxSlope$_{FL}$	-0.065230998	-0.26477002	0.1370530178	-0.061702448	-0.026585110	0.003528550	0.10326422
MaxSlope$_{FR}$	0.102231864	-0.10473614	0.3118679070	0.095713264	0.169482570	-0.006518601	0.10559377
MaxSlope$_{C}$	0.400168249	-0.02324516	0.5824301878	0.291546170	-0.140756091	-0.108622078	0.15641294
MaxSlope$_{P}$	-0.146668201	-0.31424064	0.1764542626	-0.090773398	0.350055326	0.055894804	0.12414197
MaxSlope$_{PL}$	-0.091250008	-0.27913512	0.1786096205	-0.062647830	0.252808283	0.028602178	0.11523126
MaxSlope$_{PR}$	-0.299635382	-0.44645251	0.1700839101	-0.221814706	0.992290455	0.077820675	0.16151761
MaxSlope$_{ML}$	-0.027407296	-0.20187415	0.1913708147	-0.011237712	0.085249314	0.016169584	0.10131075
MaxSlope$_{MR}$	0.010990493	-0.17827336	0.2660064494	0.041657739	0.035876327	0.030667246	0.11246286
MaxSlope$_{F-P}$	0.276242509	0.02847091	0.4287762994	0.231883067	-0.083951740	-0.044359441	0.10016073
MaxSlope$_{FL-FR}$	-0.180379780	-0.40111103	0.0526310933	-0.169621586	-0.075899104	0.010758194	0.11586469
MaxSlope$_{PL-PR}$	0.146355927	-0.10078401	0.3513549845	0.127846261	-0.041427627	-0.018509666	0.1144512
MaxSlope$_{ML-MR}$	-0.032406057	-0.17839304	0.1064192943	-0.040988132	0.143173590	-0.008582075	0.07181432
MaxSlope$_{FL-PL}$	0.023717854	-0.14445372	0.1340923012	0.003532030	-0.208879504	-0.020185824	0.07016491
MaxSlope$_{FR-PR}$	0.305190820	0.03382742	0.4464420172	0.254294959	-0.201489214	-0.050895861	0.10519168

Table S3

Complexity (*MMSE* features) pattern relevant to *gf* obtained from PLSR analysis in women (N = 62), 1LV, randomization test, $p < .05$

variable	Actual	CI 2.5%	CI 97.5%	boot.mean	Skewness	Bias	SE
AUC_{F}	-0.019810580	-0.0498996887	0.0171963141	-0.0177995883	0.195562252	0.0020109918	0.01686602
AUC_{FL}	0.029665582	0.0018218709	0.0659740512	0.0299637280	0.528607358	0.0002981462	0.01644989
AUC_{FR}	-0.029879010	-0.0535890255	0.0077860423	-0.0270044242	0.513553315	0.0028745862	0.01542252
AUC_{C}	-0.016036307	-0.0426965709	0.0245065322	-0.0132684868	0.581207160	0.0027678205	0.01690546
AUC_{P}	-0.035538631	-0.0605199762	0.0006466800	-0.0327488600	0.408213460	0.0027897712	0.01499600
AUC_{PL}	-0.028486093	-0.0553007412	0.0141840193	-0.0252682114	0.579940752	0.0032178819	0.01741940
AUC_{PR}	-0.064448251	-0.0853437101	-0.0322133494	-0.0600749523	0.284314051	0.0043732989	0.01338239
AUC_{ML}	-0.021033188	-0.0538913360	0.0136743543	-0.0198247624	0.020599056	0.0012084252	0.01701706
AUC_{MR}	0.003430915	-0.0293407611	0.0488616536	0.0050389502	0.509760176	0.0016080353	0.01998107
AUC_{F-P}	0.011212135	-0.0265035138	0.0443266454	0.0104987624	-0.212759735	-0.0007133722	0.01787452
AUC_{FL-FR}	0.064377433	0.0310213461	0.0990446924	0.0616683343	0.517325682	-0.0027090991	0.01688417
AUC_{PL-PR}	0.042233479	0.0006016148	0.0778286636	0.0398377511	-0.104922537	-0.0023957281	0.01950723
AUC_{ML-MR}	-0.024037339	-0.0698652107	0.0126138547	-0.0244839337	-0.405204242	-0.0004465949	0.02110137
AUC_{FL-PL}	0.047677651	0.0100888744	0.0791768483	0.0452157656	0.022528584	-0.0024618856	0.01724096
AUC_{FR-PR}	0.041162393	0.0004958166	0.0739584396	0.0386865498	-0.160819137	-0.0024758437	0.01857485
AVG_{F}	-0.006401572	-0.0413113169	0.0272998407	-0.0065531465	-0.076520960	-0.0001515741	0.01730558
Avg_{FL}	0.039693846	0.0080459593	0.0690269280	0.0384449815	0.117059174	-0.0012488647	0.01569618
AVG_{FR}	-0.016581595	-0.0480617729	0.0150457242	-0.0161689944	-0.005479939	0.0004126002	0.01586902
AVG_{C}	-0.009552892	-0.0362815223	0.0288543515	-0.0076944052	0.548421671	0.0018584871	0.01623951
Avg_{P}	-0.001975623	-0.0303601093	0.0386630165	-0.0004033763	0.613333823	0.0015722470	0.01743204
AVG_{PL}	-0.008581601	-0.0387010511	0.0321271370	-0.0069342196	0.444881871	0.0016473815	0.01805192
AVG_{PR}	-0.049028037	-0.0740592435	-0.0117693970	-0.0454011758	0.472668356	0.0036268609	0.01562631
AVG_{ML}	0.005774193	-0.0344474781	0.0430488159	0.0052019755	-0.059925110	-0.0005722178	0.01956663
AVG_{MR}	0.014757277	-0.0196997630	0.0563026235	0.0152957964	0.348968465	0.0005385193	0.01921597
AVG_{F-P}	-0.004342939	-0.0481821646	0.0274638203	-0.0059204098	-0.508114757	-0.0015774711	0.01926947
AVG_{FL-FR}	0.067140939	0.0354667312	0.1017644660	0.0644894890	0.618208583	-0.0026514497	0.01675778
AVG_{PL-PR}	0.042590392	0.0010901313	0.0762062461	0.0400935920	-0.182047438	-0.0024967995	0.01903711
AVG_{ML-MR}	-0.009310631	-0.0566832006	0.0251053116	-0.0105843573	-0.446302877	-0.0012737266	0.02091710
AVG_{FL-PL}	0.039945058	0.0030649751	0.0667968136	0.0368723831	-0.255216614	-0.0030726749	0.01598527
AVG_{FR-PR}	0.030033687	-0.0125029598	0.0596357415	0.0276325954	-0.436937855	-0.0024010916	0.01819673
$MaxSlope_{F}$	-0.014673499	-0.0520664316	0.0317478928	-0.0128471408	0.237815273	0.0018263581	0.02145045
$MaxSlope_{FL}$	-0.006360103	-0.0397852473	0.0343107076	-0.0052754556	0.238743510	0.0010846477	0.01911136
$MaxSlope_{FR}$	-0.016886081	-0.0445300384	0.0331255468	-0.0133068691	0.719929922	0.0035792119	0.01961382
$MaxSlope_{C}$	-0.026881738	-0.0581881022	0.0232484056	-0.0242312156	0.631004534	0.0026505226	0.02069826
$MaxSlope_{P}$	-0.036436045	-0.0642035926	-0.0003211618	-0.0337407267	0.122993544	0.0026953185	0.01635130
$MaxSlope_{PL}$	-0.030548980	-0.0593336725	0.0077899310	-0.0281396794	0.235246604	0.0024093005	0.01710759
$MaXSlope_{PR}$	-0.061721831	-0.0865206627	-0.0294904509	-0.0584462306	0.073931412	0.0032756005	0.01449188
$MaxSlope_{ML}$	-0.048891093	-0.0734104765	-0.0168924848	-0.0456560235	0.002236994	0.0032350695	0.01421261
$MaxSlope_{MR}$	0.004760853	-0.0255653906	0.0530407972	0.0072053137	0.654189357	0.0024444609	0.02011785
$MaxSlope_{F-P}$	0.008270265	-0.0390201622	0.0536958918	0.0078691892	0.008096539	-0.0004010761	0.02360219
$MaxSlope_{FL-FR}$	0.011181831	-0.0353804579	0.0412600517	0.0084267542	-0.544129967	-0.0027550765	0.01941971
$MaxSlope_{PL-PR}$	0.032514008	-0.0030596796	0.0647732640	0.0309904043	-0.069089029	-0.0015236034	0.01751580
$MaxSlope_{ML-MR}$	-0.054282972	-0.0928931956	-0.0230691402	-0.0531553721	-0.578619013	0.0011275996	0.01788107
$MaxSlope_{FL-PL}$	0.009759611	-0.0270671207	0.0466113713	0.0092913753	0.019731368	-0.0004682359	0.01893076
$MaxSlope_{FR-PR}$	0.020133264	-0.0159100116	0.0700872690	0.0217256397	0.446187040	0.0015923756	0.02207566

Note. Actual - regression coefficient, 95% CI - bias-corrected 95% bootstrapped confidence intervals, boot.mean - mean of the bootstrap, Skewness - skewness of the bootstrap distribution, Bias - estimate of bias, SE - estimate of bootstrap standard error. *Area under curve (AUC)*, obtained by trapezoidal approximation of the area delimited by the *MMSE* vector; the *AUC* feature may be viewed as the total complexity of the EEG signal represented by the *MMSE* vector; *MaxSlope*, defined as the maximum pairwise difference between first four elements (1:4 timescales) of the *MMSE* vector divided by indices' difference; the *MaxSlope* feature may be viewed as representing the maximum complexity change of the EEG signal at high-frequency fine-scales; *AvgEnt*, defined as the average value of the last four elements (9:12 timescales) of the *MMSE* vector; the *AvgEnt* feature may be viewed as representing the baseline value of entropy of the EEG signal at low-frequency coarse-scales. F – frontal, FL – frontal left, FR – frontal right, C – central, P -parietal, PL – parietal left, PR – parietal right, ML – middle left, MR – middle right.

We note that the PLS and MVDALAB packages, as used in this study, implement different PLSR algorithms: SIMPLS and BIDIAGPLS, respectively. However, the differences in the resulting PLSR models were unobservable on our data. R package implementing PLSR, the MVDALAB package (Afanador, Tran, Blanchet & Baumgartner, 2017).
